# Variation in egg size and offspring phenotype among and within seven Arctic charr morphs

**DOI:** 10.1002/ece3.9427

**Published:** 2022-10-18

**Authors:** Samantha V. Beck, Katja Räsänen, Bjarni K. Kristjánsson, Skúli Skúlason, Zophonías O. Jónsson, Markos Tsinganis, Camille A. Leblanc

**Affiliations:** ^1^ Department of Aquaculture and Fish Biology Hólar University Sauðárkrókur Iceland; ^2^ Institute of Life and Environmental Sciences University of Iceland Reykjavík Iceland; ^3^ Department of Aquatic Ecology Eawag, Swiss Federal Institute of Aquatic Science and Technology Dübendorf Switzerland; ^4^ Department of Biology and Environmental Science University of Jyväskylä Jyväskylä Finland; ^5^ Icelandic Museum of Natural History Reykjavík Iceland

**Keywords:** developmental plasticity, diversification, egg size, freshwater, maternal effects, resource polymorphism

## Abstract

Maternal effects have the potential to alter early developmental processes of offspring and contribute to adaptive diversification. Egg size is a major contributor to offspring phenotype, which can influence developmental trajectories and potential resource use. However, to what extent intraspecific variation in egg size facilitates evolution of resource polymorphism is poorly understood. We studied multiple resource morphs of Icelandic Arctic charr, ranging from an anadromous morph—with a phenotype similar to the proposed ancestral phenotype—to sympatric morphs that vary in their degree of phenotypic divergence from the ancestral anadromous morph. We characterized variation in egg size and tested whether egg size influenced offspring phenotype at early life stages (i.e., timing of‐ and size at‐ hatching and first feeding [FF]). We predicted that egg size would differ among morphs and be less variable as morphs diverge away from the ancestral anadromous phenotype. We also predicted that egg size would correlate with offspring size and developmental timing. We found morphs had different egg size, developmental timing, and size at hatching and FF. Egg size increased as phenotypic proximity to the ancestral anadromous phenotype decreased, with larger eggs generally giving rise to larger offspring, especially at FF, but egg size had no effect on developmental rate. The interaction between egg size and the environment may have a profound impact on offspring fitness, where the resulting differences in early life‐history traits may act to initiate and/or maintain resource morphs diversification.

## Graphical abstract

This study characterized egg size and development among and within seven morphs of Arctic charr that differ in their extent of phenotypic divergence from an ancestral anadromous morph. Resulting differences in early life‐history traits can have large impacts on offspring fitness and we, therefore, discuss how such fine‐scale variation in developmental processes may initiate and/or maintain phenotypic diversity in polymorphic systems.

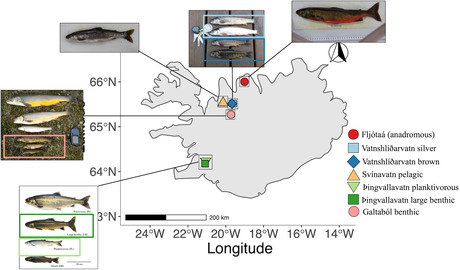

## 1 INTRODUCTION

Individual resource specialization can reduce intraspecific competition (Bolnick et al., 2003; Robinson & Wilson, 1994), leading to resource‐mediated phenotypic divergence (i.e., resource polymorphism) through amplification of various ecological, evolutionary, and developmental feedbacks that act as diversifying forces for population divergence and speciation (Levis et al., 2018; Skúlason et al., 2019; Smith & Skúlason, 1996). This process of ecological diversification can be considered along a speciation continuum of increasingly discrete phenotypic variation, from interindividual variation within a panmictic population to discrete resource morphs and, finally, reproductive isolated species (Hendry et al., 2009; Nosil, 2012; Skúlason & Smith, 1995). In addition to genetic evolutionary processes, such as mutation and drift, variation can also arise through developmental processes and thus be particularly strong at early life stages (West‐Eberhard, 2003). Our understanding of how variation is generated and maintained is slowly increasing as the deeply intertwined nature of evolution and development is realized (Hendrikse et al., 2007; Minelli, 2015; Moczek et al., 2015; Skúlason et al., 2019).

Maternal effects, such as egg size, can act as an important source of early life stage phenotypic variation. The classic Smith and Fretwell (1974) egg size model suggests offspring fitness increases with increasing propagule size. As a result, mothers face a trade‐off between increasing offspring fitness at a cost of a reduction in fecundity, which is a function of female size (Einum & Fleming, 2000). However, rather than the evolution of a single‐optimum egg size, variable egg size may be favored (e.g., via bet‐hedging, Slatkin, 1974) and hence influence between and within female variation in egg size (e.g., Bernardo, 1996b; Johnston & Leggett, 2002; Koops et al., 2003; Marshall et al., 2008). Females may thus ‘hedge their bets’ in stochastic environments to maximize fitness (Hutchings, 1997; Koops et al., 2003; Marshall et al., 2008), which can consequently alter developmental processes and associated phenotypic outcomes (Bernardo, 1996a; West‐Eberhard, 2003), as seen in many taxa (e.g., *Drosophila* [Eizadshenass & Singh, 2015]; fish [Cogliati et al., 2018; Kinnison et al., 2001]; amphibians [Pfennig & Martin, 2009; Räsänen et al., 2005]; and birds [Badyaev, 2008]). For example, differences in embryo size has been found to influence the expression of genes involved in growth and skeletal development (Beck et al., 2019), as well as offspring phenotype (Beck et al., 2020) in a single morph of Arctic charr (*Salvelinus alpinus*) that has large variation in egg size. However, no study to date has examined how variation in egg size changes across multiple wild polymorphic populations that differ in their degree of phenotypic divergence from a proposed ancestral type.

The depauperate nature of postglacial lakes in the Northern hemisphere provides a well‐suited opportunity to examine recent evolutionary diversification within a species (Skúlason et al., 2019). Lakes were colonized by anadromous fishes after the last glaciation, and have since occupied vacant niches, evolving through resource polymorphism from anadromy to varying degrees of specialized freshwater morphs (Skúlason & Smith, 1995; Snorrason & Skúlason, 2004). Such systems enable comparisons in divergence between several sympatric specialized morphs and the putative ancestral populations (i.e., the ancestral ‘stem’; Doenz et al., 2019; Levis & Pfennig, 2016; Parsons et al., 2011; West‐Eberhard, 2003; Wund et al., 2008). In Arctic charr, egg size can be highly variable among and within females (Baroudy & Elliott, 1994; Lasne et al., 2018; Leblanc et al., 2016; Wallace & Aasjord, 1984), as well as among morphs (e.g., Smalås et al., 2017). Such variation can contribute toward alternative feeding behavior (Benhaïm et al., 2003; Leblanc et al., 2011, 2016), developmental rates (Eiríksson et al., 1999; Leblanc et al., 2011), body size (Leblanc et al., 2016), gene expression patterns (Beck et al., 2019), and craniofacial shape (Beck et al., 2020). Here, we study seven morphs of Icelandic Arctic charr that vary in magnitude of phenotypic and genetic divergence, including an ancestral anadromous morph. We propose that egg size is associated with the development of morph‐specific traits. Specifically, we hypothesize that: (i) egg size will differ among morphs, as life‐history theory predicts that environmental selection should favor an optimum propagule size (Smith & Fretwell, 1974), and the difference in mean egg size would be largest between morphs that are more phenotypically diverged from the ancestral anadromous morph due to increased specialization on alternative resources; (ii) variation in egg size among females within morphs is smaller in more phenotypically diverged morphs due to the more predictable environments that they have adapted towards (Koops et al., 2003); and finally, (iii) the timing and size at which offspring reach certain stages of early development differ among morphs as a result of adaptation to local ecological conditions, and is correlated with egg size (Gillooly et al., 2002).

## 2 METHODS

### 2.1 Study system

**FIGURE 1:**
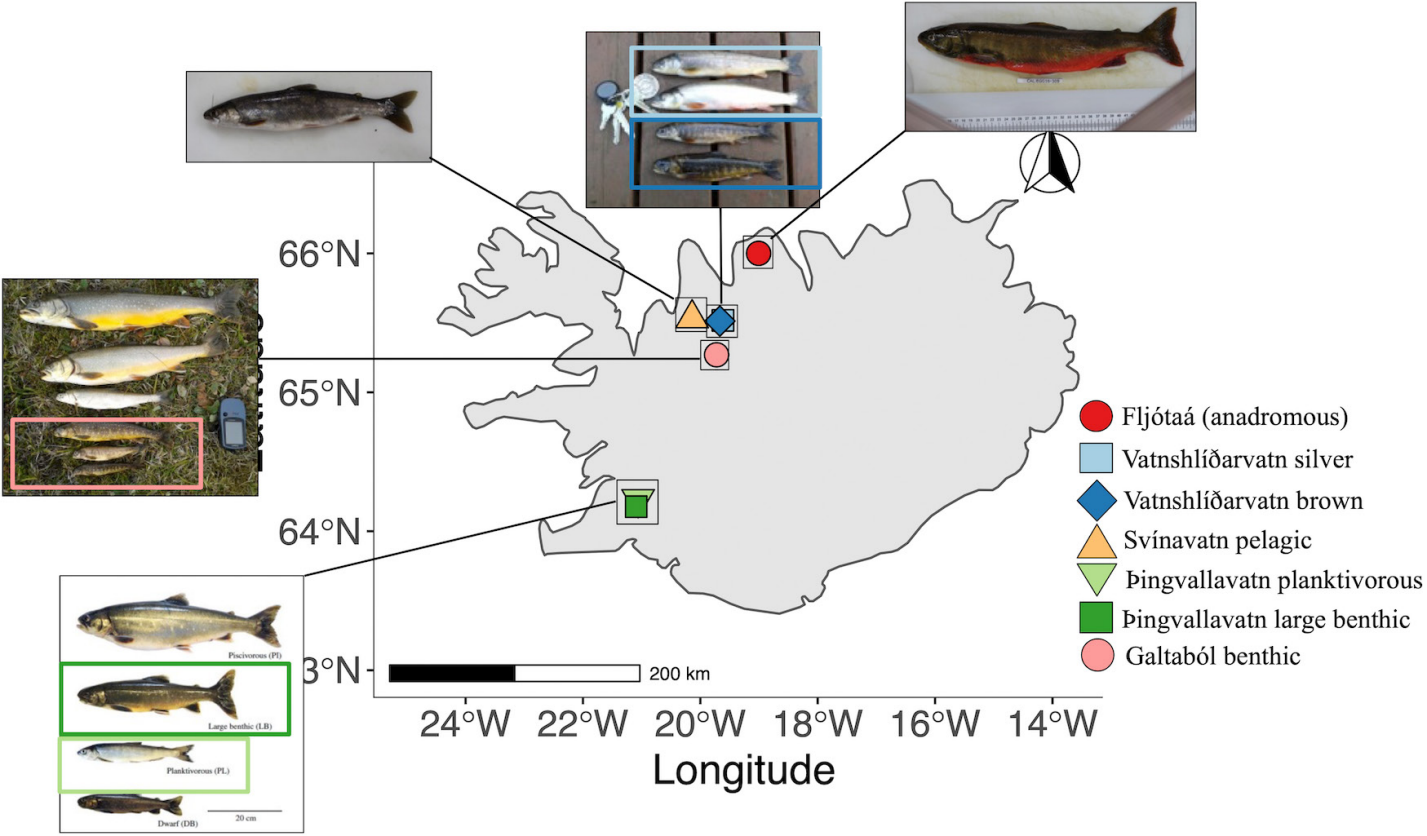
Map of Iceland with sampling locations for seven Arctic charr morphs (Fljótaá, Vatnshlíðarvatn silver, Vatnshlíðarvatn brown, Svínavatn, Þingvallavatn pelagic, Þingvallavatn large benthic, and Galtaból benthic), indicated by different symbols and colors. In cases where multiple sympatric morphs occur, only those used in this study are highlighted. All morphs spawn within lakes except for Vatnshlíðarvatn silver (which migrates to inlet and outlet streams to spawn) and the anadromous Fljótaá. Image of morphs from lake þingvallavatn were modified from Johnston (2004). Sympatric morphs share similar colors.

We studied a total of seven Arctic charr morphs (Figure 1; see Table 1 for details on lakes and system characteristics) based on their phenotypic proximity to the ancestral anadromous morph (e.g., pelagic and migratory life histories, as well as body shape and size) and ordered accordingly: (1) The anadromous morph from the river Fljótaá (FJ) was used as a proxy of an ancestral phenotype; (2) Two morphs from lake Vatnshlíðarvatn (silver, VS, and brown, VB), with VS retaining its migratory life‐history strategy by spawning in the inlets and outlets of the lake but has a smaller size than the anadromous morph, whilst VB is smaller and has a more specialized benthic diet (*Eurycercus* sp. is common prey) and spawns within the lake. The phenotypic and genetic divergence between the two morphs is subtle (Brachmann et al., 2021; Gíslason et al., 1999), likely reflecting the physically simple, small, and shallow nature of lake Vatnshlíðarvatn (Jónsson & Skúlason, 2000); (3) A pelagic morph from lake Svínavatn (SV), a large, deep lake, which harbors three putative Arctic charr morphs: a pelagic planktivorous morph (studied here) which spawns within the lake and is smaller than the anadromous morph, a piscivorous and a benthic morph (Gíslason et al., 1999). SV differs from the other two morphs both phenotypically and genetically (Brachmann et al., 2021;Gíslason et al., 1999; Wilson et al., 2004); (4) Two of the four Arctic charr morphs from Icelands' largest natural lake, Þingvallavatn: the pelagic planktivorous (TP) and large benthic (TLB) morph. Both morphs diverge strongly from the anadromous morph, with TP having a smaller size and only feeds on a planktivorous diet, whilst TLB has a large body size (similar to the anadromous) and lives and feeds exclusively in the benthic environment, with associated phenotypic specializations (blunted snout and subterminal jaw). These strong phenotypic differences between TP and TLB occur very early in development (Kapralova et al., 2015), reflecting their trophic specializations (Malmquist et al., 1992), and genetic differentiation (Brachmann et al., 2021); and finally (5) a small benthic morph from lake Galtaból (GB). Galtaból is a small and remote highland lake, which harbors two sympatric morphs that are strongly phenotypically and genetically diverged from each other: the small benthic morph (studied here) and a large piscivorous morph (Gíslason et al., 1999; Wilson et al., 2004). The small size, specialized benthic diet, and associated phenotype makes the small benthic the most diverged morph from the anadromous morph out of all morphs studied here. In addition, the two morphs in lake Galtaból are reproductively isolated and are considered one of the very few examples of true sympatric speciation (Brachmann et al., 2021; Coyne & Orr, 2004; Gíslason et al., 1999). Not all sympatric morphs inhabiting each lake could be included due to limited knowledge on spawning location and timing, or because too few individuals were caught.

**TABLE 1:** Lake and system characteristics for sampled Arctic charr (*Salvelinus alpinus*) populations in Iceland.

	Fljótaá (river)	Vatnshlíðarvatn (lake)	Svínavatn (lake)	Þingvallavatn (lake)	Galtaból (lake)
Total no. of morphs	1 (anadromous)	2 (silver and brown)	3 (1 benthic and 2 pelagic)	4 (2 pelagic and 2 benthic)	2 (large pelagic, small benthic)
Morph(s) sampled and acronym	1 (anadromous)	2 (silver and brown)	1 (pelagic planktivorous)	2 (planktivorous and large benthic)	1 (small benthic)
	FJ^a^	VS^a^ and VB	SV	TP and TLB	GB
Latitude	66.000192	65.516745	65.526436	64.191946	65.2654722
Longitude	−19.007276	−19.628921	−20.052359	−21.136087	−19.7299389
Size	8 km long	70 ha	1200 ha	8400 ha	140 ha
Max. depth	Variable	6 m	38.5 m	114 m	10 m
Elevation	–	280 m	123 m	100 m	450 m
Substrate	Rocky	Muddy but gravel in shallows	Rocky	Volcanic rocky	Muddy but gravel/rocks in shallows
Other fish sp.	Atlantic salmon	No	Brown trout	Brown trout	Three‐spined stickleback
				Three‐spined stickleback	
Within pair genetic divergence (*F*_ST_)	–	0.05	SV/benthic = 0.18	TP and TLB = 0.15	GB/large pelagic = 0.43
			SV/pisc. = 0.0007	TP/small benthic = 0.13	
				TLB/small benthic = 0.08	

^a^ Adults spawn in streams.

### 2.2 Crosses and maternal phenotype

Fish from all morphs were caught at the end of summer and/or in autumn between 2014 and 2016: FJ = 2015, 2016; VB = 2014, 2015, VS = 2014, 2015; SV = 2015, 2016; TLB = 2014, 2015; TP = 2014:2016; GB = 2014, 2015. Sampling over multiple years was necessary due to limitations on sampling times and locations, as well as handling and rearing of juveniles. Gill nets and electrofishing were used to collect fish that spawned in lakes and streams, respectively. Female size and egg size data were measured on wild collected females (see below). Fecundity data were not included due to some females being partially spent (i.e., having released some of their eggs).

To study the effects of egg size on early life stage phenotypic variation, we conducted laboratory rearing. Mature females (*N* = 14 (VS) to 24 (TP)) from each morph (Table 2; Table S1) were stripped and eggs and milt mixed in the field. In most cases, the same male was used to fertilize multiple females (typically 2–4; Table S1) to minimize genetic variation between offspring. However, a small proportion of females were mated to a male not shared with other females (Table S1). This design causes variation in the relatedness of the offspring (half‐sibs vs. full‐sibs) and does not allow us to fully disentangle direct genetic effects from maternal effects, but was used to minimize unsuccessful crosses. Fertilized eggs were allowed to water‐harden before transport to Hólar University's aquaculture facilities in Verið, Sauðárkrókur. After stripping, males and females were sacrificed with a sharp blow to the head and weighed in the field to the nearest 0.1 g, before being brought back to the laboratory where fork length (FL) was measured to the nearest 0.1 cm and two sagittal otoliths removed. For reading, otoliths were immersed in 96% ethanol to increase clarity of the annuli for age determination. Otoliths were photographed under reflected light against a dark background using a Canon 600D camera mounted on a Leica MZ12 stereomicroscope and images digitally enhanced using ImageJ v1.48 (Schneider et al., 2012) according to Campana and FAO (2014). Two readers read the otoliths to ensure accurate aging, with discrepancies revisited and clarified where possible, or removed if no agreement. Otoliths were also read twice by each reader under a microscope and twice using randomized digital images to reduce bias (Tsinganis, 2016). It must be noted that using whole otoliths may underestimate older age classes in Arctic charr, however, the age at which this occurs may vary between populations (e.g., Gallagher & Wastle, 2021).

**TABLE 2:** Sampling design, measurements of female and offspring traits, and developmental timing (degree days; DD) in seven morphs of Arctic charr (*Salvelinus alpinus*) found in allopatry and sympatry throughout Iceland.

	FJ	VS	VB	SV	TP	TLB	GB
Total no. of families used for each variable							
Egg size (mm)	15	14	17	17	24	17	15
DD	15	6	10	0	10	14	0
Female measurements							
*N* female	15	14	17	17	24	17	15
Mean age (years)	5 ± 1.3	6 ± 0.8	6 ± 1.5	5 ± 1.3	6 ± 1.0	9 ± 2.2	6 ± 0.9
Mean FL (cm)	37.7 ± 3.87	24.4 ± 1.87	18.0 ± 3.84	25.6 ± 1.59	20.7 ± 1.28	36.0 ± 5.27	21.8 ± 1.88
Egg size (PF + E)							
*N* families	15	14	16	8	22	15	14
Mean size (mm)	4.5 ± 0.20	4.5 ± 0.23	4.4 ± 0.49	5.3 ± 0.22	5.0 ± 0.16	5.1 ± 0.19	5.1 ± 0.18
CV (%)	6.1	5.9	12.1	5.5	5.6	5.1	4.1
*N* off.	810	476	640	367	483	788	334
PF DD	10 ± 0.49	11 ± 1.04	22 ± 7.39	10 ± 0.49	10 ± 4.20	14 ± 1.88	11 ± 0.53
E DD	201 ± 2.75	219 ± 2.71	228 ± 8.14		227 ± 24.33	212 ± 6.59	
Hatching							
*N* families	15	14	15	4	24	12	4
Mean length (mm)	14.8 ± 1.09	14.3 ± 1.08	14.6 ± 1.27	15.4 ± 1.15	16.7 ± 0.96	15.4 ± 1.46	14.8 ± 0.73
CV (%)	7.4	7.6	8.7	7.5	5.7	9.5	5
*N* off.	429	221	245	63	277	169	74
DD	417 ± 26.30	414 ± 22.40	448 ± 26.90	419 ± 24.70	447 ± 14.10	443 ± 12.90	440 ± 9.71
First feeding							
*N* families	15	14	15	4	23	14	12
Mean length (mm)	20.2 ± 0.10	18.7 ± 0.99	17.8 ± 1.85	22.8 ± 1.40	21.1 ± 1.16	20.5 ± 1.11	19.8 ± 1.64
CV (%)	4.9	5.3	10.4	6.2	5.5	5.4	8.3
*N* off.	359	273	283	38	313	280	124
DD	650 ± 27.50	637 ± 8.92	677 ± 28.60	727 ± 24.90	647 ± 21.90	665 ± 20.40	619 ± 0.64

*Note*: The seven morphs are FJ, Fljótaá (ancestral anadromous); VS, Vatnshlíðarvatn silver; VB, Vatnshlíðarvatn brown; SV, Svínavatn; TLB, Þingvallavatn large benthic; TP, Þingvallavatn planktivorous; and GB, Galtaból benthic. Egg size (diameter, mm; ±standard deviation) was taken from measurements at both postfertilization (PF) and eye stage (E) for each family (i.e., two measurements per family). *N*, sample size. Mean fork length (FL; cm); Mean length, standard length of embryos (mm).

Abbreviations: CV, coefficient of variation; off., offspring.

### 2.3 Rearing of embryos and sample collection

In the laboratory, eggs were reared in family groups in common‐garden conditions, as described by Beck et al. (2019). Eggs from a given family were split between several cages when eggs were numerous (*n* = >100) to ensure sufficient oxygenation and comparable density in each cage (i.e., two or three layers of eggs; Table S1). Eggs were reared at a mean temperature of 4.25°C ± 0.48 standard deviations (SD) and developmental timing was tracked with an accumulative temperature estimate (degree days, DD; Pruess, 1983; Table S1). To characterize variation in size and developmental times, morphs were sampled at four points during development, as detailed in Beck et al. (2019): (1) postfertilization (PF), (2) eye stage (E), when eye lenses are formed and retinas pigmented, (3) hatching (H), when individuals have hatched but still rely on nutrition from the yolk sac (i.e., ‘free embryos,’ Flegler‐Balon, 1989), and (4) first feeding (FF), when individuals initiate exogeneous feeding (Ballard, 1973). Once approximately 50% of individuals within a given family had reached a particular stage, those individuals within that stage were sampled and the number of DDs used as a measure of developmental time per family (Leblanc et al., 2011).

Mean egg size per female (*N* > 15 eggs) was estimated by measuring egg diameter of embryos at PF and E stages (*N* = 3898). To obtain a broad coverage of the full egg size range within each female, as well as ensuring that the extremes were sampled, the same person visually selected and removed ~25% of a female's eggs (where possible) for estimates of egg size (PF and E) by selecting equal proportions of small, medium, and large eggs (Table S1; Benhaïm et al., 2003; Leblanc et al., 2011). Average measurements of offspring size and DD were taken for each female at H (size: total *N* offspring = 1478, *N* offspring per female = 5–80; DD: total *N* offspring = 870, *N* offspring per female = 5–80) and FF (size: total *n* offspring = 1670, *N* offspring per female = 6–45; DD: total *N* offspring = 985, *N* offspring per female = 9–45; see Table S1 for further details on sample sizes). All individuals (eggs, or left‐side of H and FF embryos) were digitally photographed (Canon EOS 650D, 100 mm macro lens) with a scale. Embryo sizes (average of 4 egg diameters for PF and E and standard length for H and FF offspring) were measured from photos to the nearest 0.01 mm (Leblanc et al., 2016) using the program Fiji (Schindelin et al., 2012). Differences in number of females for which offspring were sampled across developmental stages arose due to offspring mortality or sampling error. In particular, GB and SV had small sample sizes at H (female *N* = 4) and at FF (SV, female *N* = 4). In all other cases, offspring from a minimum of 11 females per morph were sampled at each developmental stage (Table 2).

### 2.4 Statistical analyses

All statistical analyses were conducted on female means in R (R Core Team, 2021). All model residuals were investigated using plots and histograms to test for normality and heteroskedasticity. Given that morphs, female size and age were relatively confounded (see Section 3), we compared alternative models using the Akaike Information Criterion (AICc) to determine the best fitting predictor (i.e., female FL, female age, or morph) for each response variable where appropriate.

### 2.5 Female phenotype

The relationship between female FL and age was tested using a linear model, with FL as the response variable and age as a continuous predictor, to determine whether both variables needed to be included for downstream analyses. Two separate ANOVAs were then performed to determine the effect of morph on: (1) female FL and (2) female age, with morph as a fixed factor. Comparisons of trait means were conducted using least square means (LSM) from the *lsmeans* package (Lenth, 2016).

### 2.6 Egg size differences

To determine the best fitting predictor for mean egg size out of the three correlated factors (female FL, age, and morph), we conducted model comparisons that included each predictor in a separate model. The effect of female FL or age (both continuous predictors) on mean egg size was examined using linear models, whilst an ANOVA was used to determine morph differences in mean egg size. To measure egg size variation within females, we calculated the coefficient of variation per female (CV_eggSize_ = SD egg size/mean egg size). Differences in egg size and egg size variation among morphs were plotted using LSM.

### 2.7 Egg size effects on offspring phenotype

To test for the effect of morph and female mean egg size on offspring phenotype (developmental time and size at both H and FF), we used separate factorial linear models within each developmental stage (H and FF). We first analyzed differences among morphs in developmental time (i.e., DD to a given stage) to H or FF, using an ANOVA with morph as a fixed factor. Next, we included morph × egg size interaction to test for effects of egg size and egg size slope heterogeneity for offspring size at both H and FF using an ANCOVA with morph as a fixed factor and egg size as a covariate. Nonsignificant interactions were dropped from models and subsequent comparisons of trait means were conducted using LSM. Note that for analyses of developmental time, only a subset of females could be used due to sampling error (Table 2), and SV and GB morphs were not included due to small sample sizes.

## 3 RESULTS

### 3.1 Size and age of females across morphs

**FIGURE 2:**
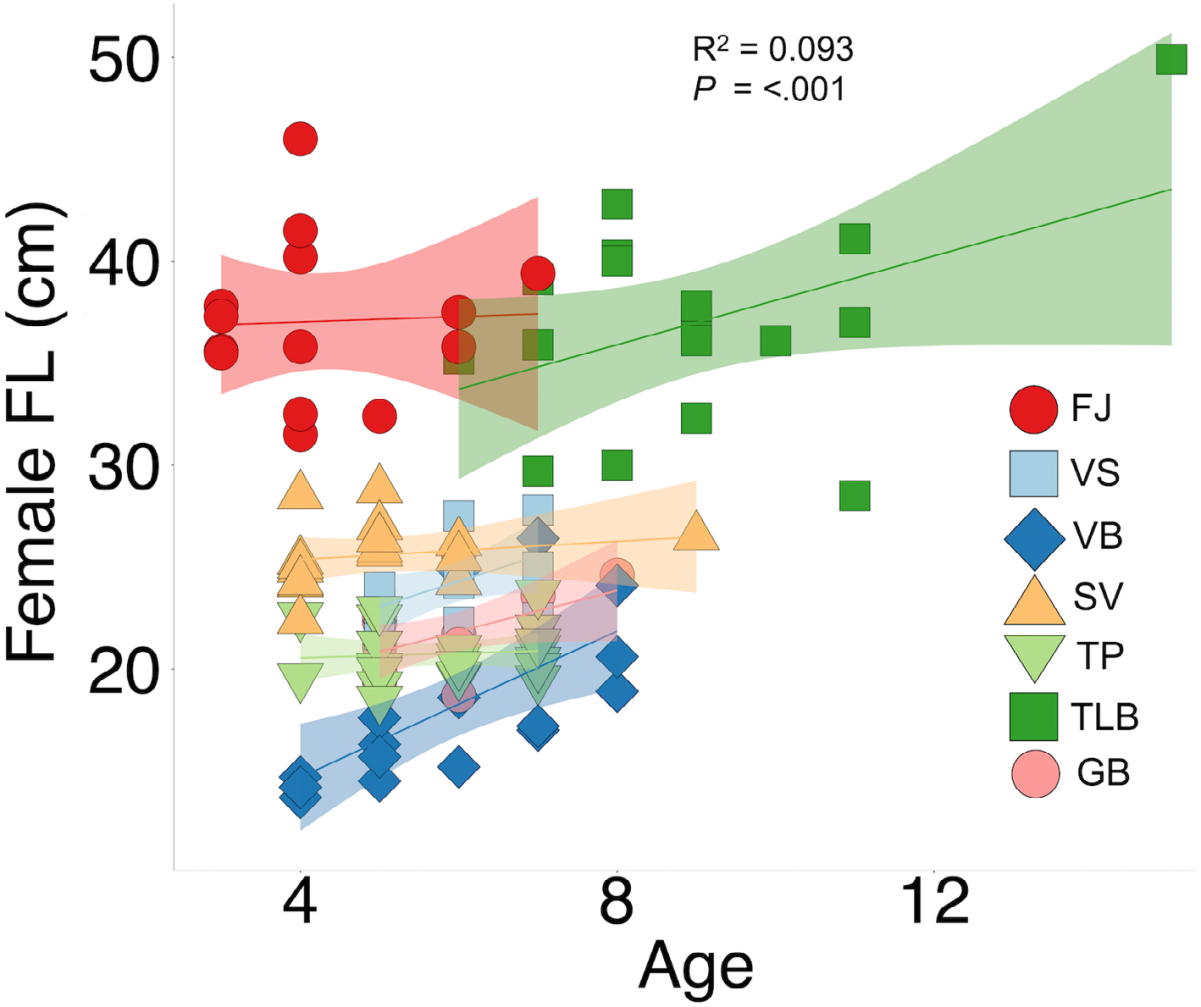
Relationship between female size (fork length, cm) and age (year) in seven morphs of Icelandic Arctic charr (FJ, Fljótaá; VS, Vatnshlíðarvatn silver; VB, Vatnshlíðarvatn brown; SV, Svínavatn; TP, Þingvallavatn pelagic; TLB, Þingvallavatn large benthic; and GB, Galtaból benthic). Different colors and symbols represent each morph, which are ordered according to phenotypic proximity to the ancestral anadromous morph (FJ). Sympatric morphs share similar colors. Strength and significance of relationship indicated by adjusted *R* ^2^ and associated significance. Shaded areas represent 95% confidence intervals.

Female FL and age were significantly, albeit weakly, correlated (*F*_1,116_ = 13.02, *R* ^2^ = .093, *p* < .001), with older females being the largest (Figure 2). Female size differed between morphs and morph was a better predictor of female FL than age (*F*_6,112_ = 102.95, *p* < .0001, Table 3). The largest females were those from FJ and TLB (all pairwise comparisons <.0001), with females reaching 46 cm and 49.9 cm, respectively. The smallest females originated from VB and TP, with sizes as small as 13.7 cm and 18.5 cm, respectively (all pairwise comparisons <.0001, apart from GB females, which did not differ in size from TP females; Figure S1). Morphs also differed in age (*F*_6,112_ = 20.01, *p* < .0001), with females from FJ being the youngest (minimum of 3 years old) in comparison to all morphs except SV (pairwise comparisons <.05) and TLB females being the oldest (maximum of 15 years old; all pairwise comparisons <.0001).

**TABLE 3:** Model comparisons using the Akaike information criterion (AICc) in cases where variables were confounded.

Response	Predictor	AICc	Delta AICc	AICcWt	LL	Adj. *R* ^2^
Female FL	**Morph**	**615.69**	**0**	**1**	**−299**	**.838**
	Age	808.21	192.52	0	−401	.093
Age	**Morph**	**411.77**	**0**	**1**	**−197**	**.494**
	Female FL	474.62	62.85	0	−234	.093
Egg size	**Morph**	**25.95**	**0**	**1**	**−4**	**.603**
	Age	122.63	96.69	0	−58	.058
	Female FL	129.25	103.3	0	−62	.005
CV_eggSize_	**Morph**	**−626.15**	**0**	**1**	**322**	**.220**
	Female FL	−604.73	21.42	0	305	.018
	Age	−596.7	29.45	0	301	−.007

*Note*: Delta AICc, difference in AICc between this model and the best model; AICcWt, indicates the levels of support (or weight) of the model.

Abbreviations: LL, Log‐likelihood; Adj. *R* ^2^, adjusted *R* ^2^.

Bolds indicates the best model.

### 3.2 Egg size differences among morphs

**FIGURE 3:**
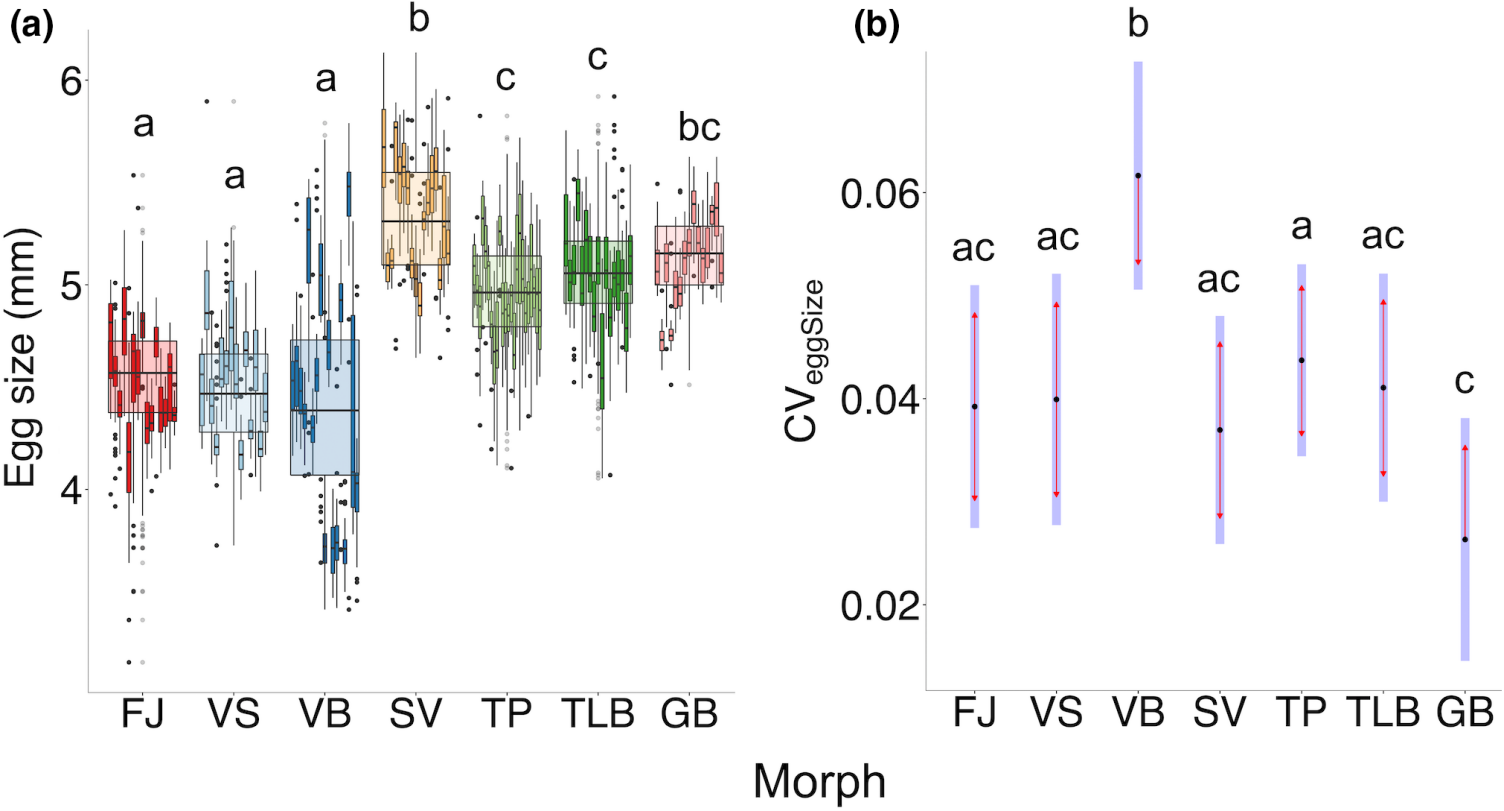
Pairwise comparisons of least‐square means showing how seven morphs of Icelandic Arctic charr (FJ, Fljótaá; VS, Vatnshlíðarvatn silver; VB, Vatnshlíðarvatn brown; SV, Svínavatn; TP, Þingvallavatn pelagic; TLB, Þingvallavatn large benthic; and GB, Galtaból benthic) differ in: (a) egg size, where individual boxplots represent each female, whilst overlaid grouped boxplots show differences at a morph level, with outliers represented as points. Morphs are indicated by different colors, with sympatric morphs sharing similar colors; and (b) least square means (LSM) of egg size variation (coefficient of variation, CV_eggSize_), whereby shaded bars represent confidence intervals of LSM and red arrows enable comparisons among them and associated significance indicated using letters (*p* < .05). All morphs are ordered according to phenotypic proximity to the ancestral anadromous morph (FJ).

As morph and FL were confounded, we ran models with morph and FL separately to test for (a) differences among morphs and (b) relationship between FL and egg size. Morph predicted mean egg size better than did female age or FL (Table 3), with mean egg size differing among morphs (*F*_6,112_ = 30.82, *p* < .0001; Figure 3a). Mean egg size ranged from 3.72–5.64 mm, with FJ, VS, and VB having smaller eggs compared to all other morphs (pairwise comparisons: *p* < .0001; Figure 3a). VB had the smallest eggs (mean ± SD: 4.4 ± 0.49 mm), whilst the SV morph had the largest eggs (5.3 ± 0.22 mm). Morph was also the best fitting predictor of egg size variation (CV_eggSize_) among females (Table 3). CV_eggSize_ differed among morphs (*F*_6,112_ = 6.53; *p* < .0001) with VB having the highest CV_eggSize_ (12.1%; *p* < .0001), and GB having lower CV_eggSize_ (4.1%) than TP (5.6%; *p* < .05; Figure 3b).

**FIGURE 4:**
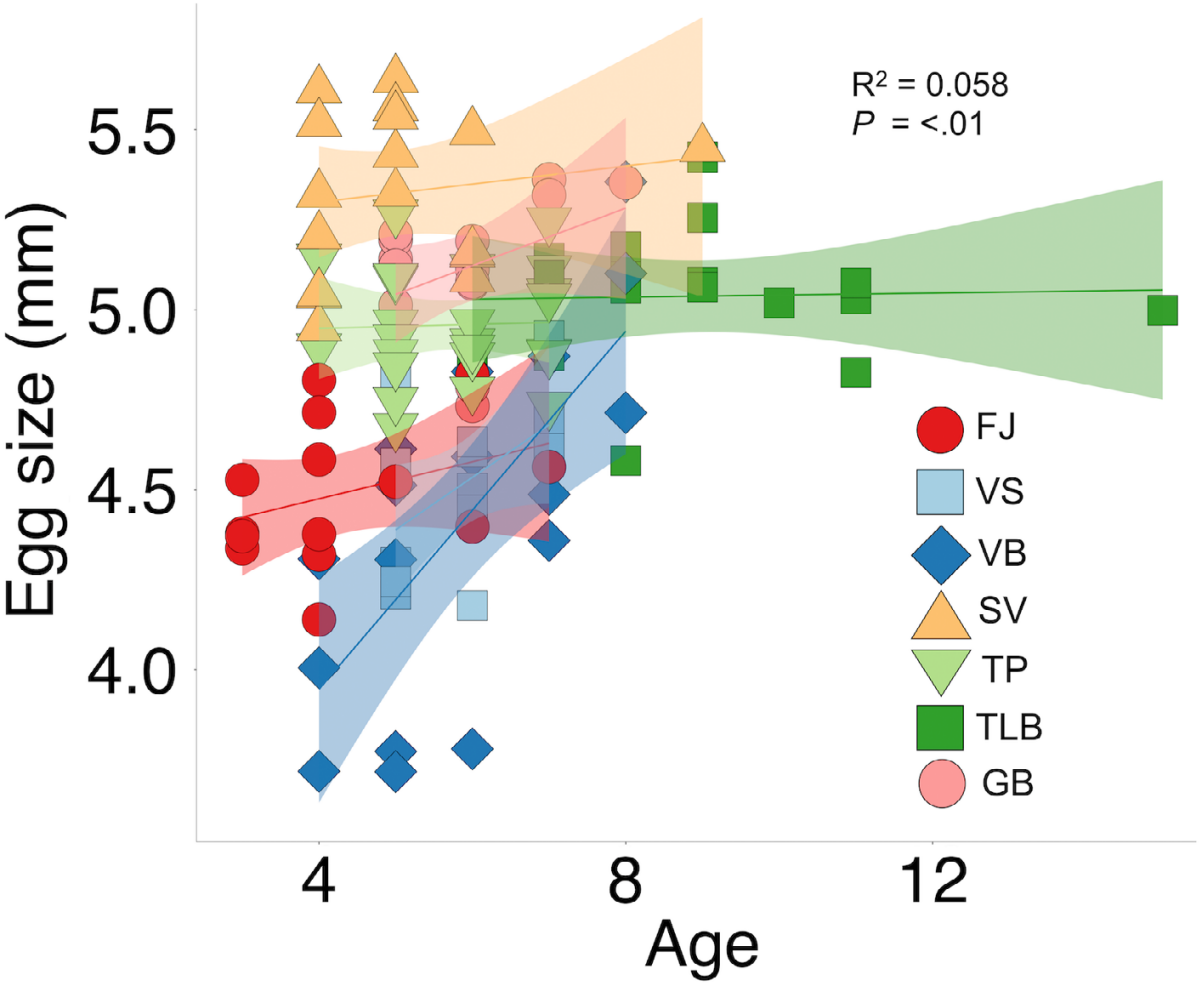
Relationship between egg size and female age in seven morphs of Icelandic Arctic charr (FJ, Fljótaá; VS, Vatnshlíðarvatn silver; VB, Vatnshlíðarvatn brown; SV, Svínavatn; TP, Þingvallavatn pelagic; TLB, Þingvallavatn large benthic; and GB, Galtaból benthic), indicated by different colors and symbols. Sympatric morphs share similar colors and are ordered according to phenotypic proximity to the ancestral anadromous morph (FJ). Strength and significance of relationship indicated by adjusted *R* ^2^ and associated significance. Shaded areas represent 95% confidence intervals.

Egg size was not correlated with female FL (*F*_1,117_ = 1.60, *p =* .209; Figure S2) but did correlate with female age (*F*_1,117_ = 8.17, *p* = .005), whereby older females generally produced larger eggs (Figure 4). Neither female FL nor age had an effect on egg size variation (Table 4).

**TABLE 4:** Linear models used to test the effect of morph on female fork length (FL), egg size, as well as on offspring traits (time taken to hatching and first feeding stage, as well as size at hatching and first feeding) in seven different morphs of Icelandic Arctic charr (FJ, Fljótaá; VS, Vatnshlíðarvatn silver; VB, Vatnshlíðarvatn brown; SV, Svínavatn; TLB, Þingvallavatn large benthic; TP, Þingvallavatn planktivorous; GB, Galtaból benthic).

Response variable	*N*	Factor	Sum Sq	DF	*F*	*p*	*β*	SE	*t*	*p*
(a) Female phenotype										
Female FL	119	**Morph**	**5867.20**	**6**	**102.95**	**<.0001**				
		*Residuals*	1063.80	112						
	119	**Age**	**694.00**	**1**	**13.02**	**.000**				
		*Residuals*	6182.20	116						
Age	119	**Morph**	211.48	6	20.01	**<.0001**				
		*Residuals*	195.51	111						
(b) Absolute egg size										
Egg size	119	**Morph**	**12.37**	**6**	**30.82**	**<.0001**				
		*Residuals*	7.49	112						
	119	Female FL	0.27	1	1.60	.209				
		*Residuals*	19.59	117						
	119	**Age**	**1.30**	**1**	**8.17**	**.005**				
		*Residuals*	18.53	116						
CVeggSize	119	**Morph**	**0.01**	**6**	**6.53**	**<.0001**				
		*Residuals*	0.03	112						
	119	Female FL	0.00	1	3.17	.077				
		*Residuals*	0.04	117						
	119	Age	0.00	1	0.24	.623				
		*Residuals*	0.04	116						
(c) Offspring traits										
Time of H	43	**Morph**	14634.70	4	24.53	**<.0001**				
		*Residuals*	5667.80	38						
	45	**Morph**	**14337.00**	**4**	**23.44**	**<.0001**				
		Egg size	9.50	1	0.06	.804				
		*Residuals*	5658.30	37						
Size at H	45	**Morph**	**66.83**	**6**	**14.96**	**<.0001**				
		*Residuals*	61.81	83						
	45	**Morph**	**42.02**	**6**	**9.61**	**<.0001**				
		Egg size	2.02	1	2.77	.100				
		*Residuals*	59.79	82						
Time of FF	45	**Morph**	5689.60	4	3.45	.016				
		*Residuals*	16494.50	40						
	45	**Morph**	**5150.60**	**4**	**3.26**	**.021**				
		Egg size	1089.30	1	2.76	.105				
		*Residuals*	15405.20	39						
Size at FF	45	**Morph**	**177.65**	**6**	**33.37**	**<.0001**				
		*Residuals*	79.85	90						
	45	**Morph**	75.23	6	23.23	**<.0001**				
		**Egg size**	**25.56**	**1**	**47.35**	**<.0001**				
		Egg size * Morph	**9.50**	**6**	**2.93**	**.012**				
		*Residuals*	44.80	83						
		**FJ**					**2.82**	**1.00**	**2.81**	**.006**
		VS					1.33	0.90	1.47	.145
		**VB**					**1.54**	**0.38**	**4.08**	**.000**
		**SV**					**6.58**	**2.41**	**2.73**	**.008**
		TP					1.79	0.97	1.84	.069
		TLB					1.68	1.16	1.45	.151
		**GB**					**5.96**	**1.19**	**5.03**	**<.0001**

*Note*: Degrees of freedom for all slopes = 83. *N*, number of individuals used.

Abbreviations: CV_eggSize_, coefficient of variation in egg size; FF, first feeding; H, hatching.

Significant effects are in bold.

### 3.3 Egg size and offspring phenotype

**FIGURE 5:**
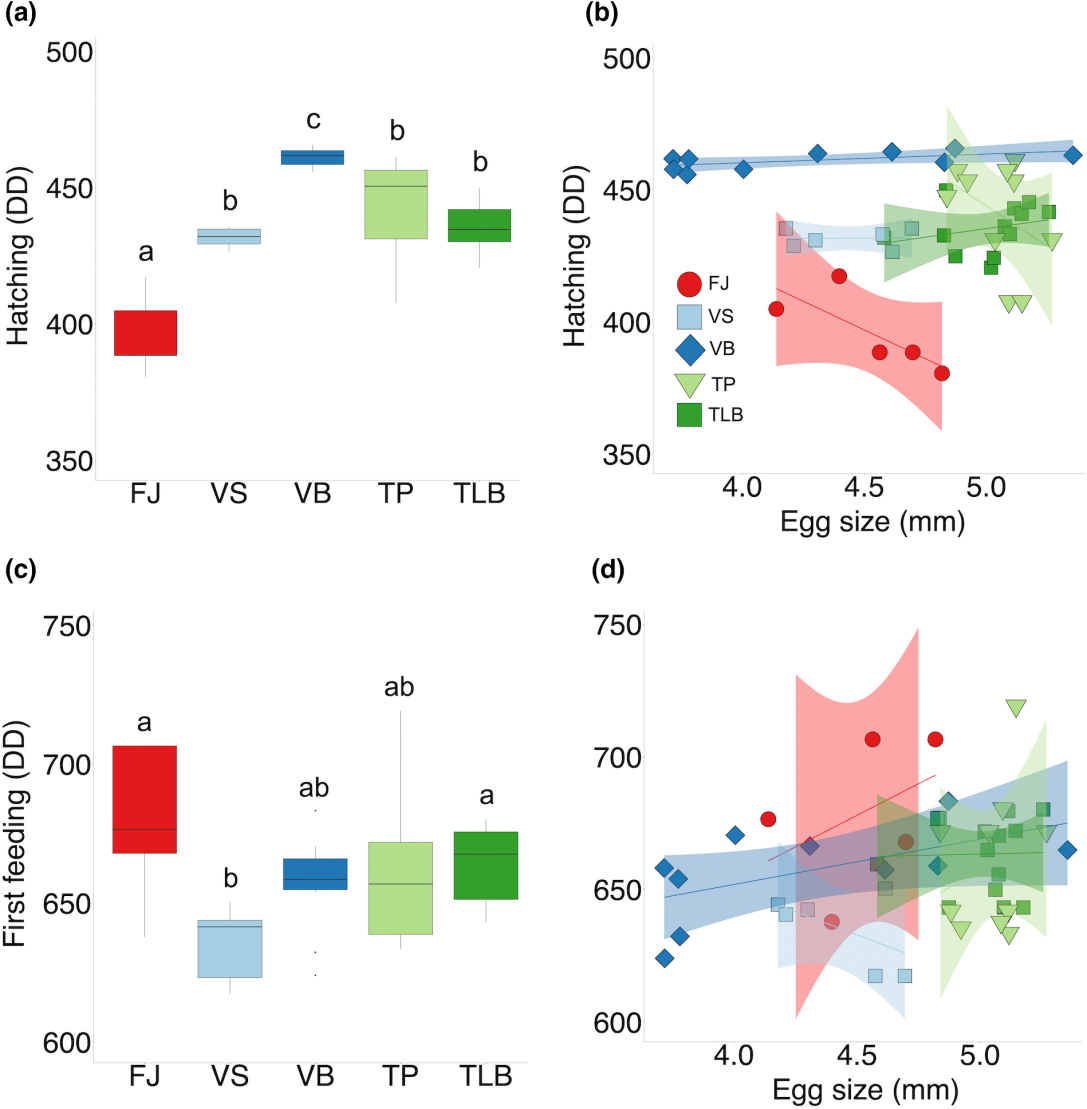
Developmental time in degree days (DD) for seven morphs of Icelandic Arctic charr (FJ, Fljótaá; VS, Vatnshlíðarvatn silver; VB, Vatnshlíðarvatn brown; SV, Svínavatn; TP, Þingvallavatn pelagic; TLB, Þingvallavatn large benthic; and GB, Galtaból benthic) to reach: (a) hatching and (b) the relationship with egg size; and (c) first feeding and (d) the relationship with egg size. Morphs from the same lake share similar colors and are ordered according to phenotypic proximity to the ancestral anadromous morph (FJ). Letters indicate significant differences (*p* < .05; a, c). Each morph has their own symbol and shaded areas represent 95% confidence intervals (b, d).

Morphs differed in time to H (*F*_4,40_ = 25.51, *p* < .0001), with FJ offspring hatching earlier (DD = 396) and VB later (DD = 461) than offspring of all other morphs (all Tukey's pairwise *p* < .01; Figure 5a). There was no effect of egg size on time to H (Table 4; Figure 5b). Morphs also differed in time to FF (*F*_4,40_ = 3.45, *p* = .016), with VS feeding earlier (DD = 635) than FJ (DD = 679) and TLB (DD = 663; all Tukey's pairwise: *p* < .05; Figure 5c) individuals. There was no significant effect of egg size on time to FF (Table 4; Figure 5d).

**FIGURE 6:**
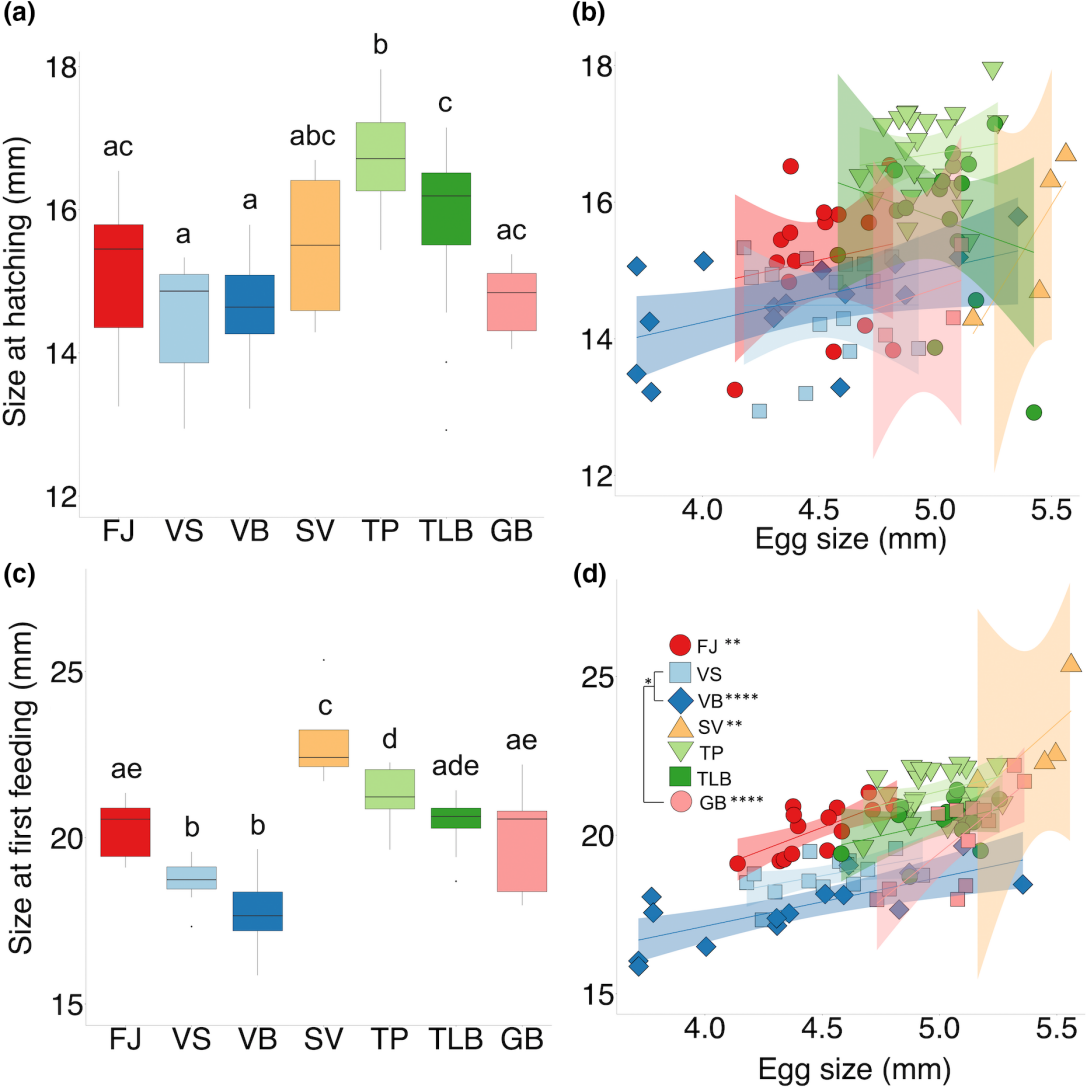
Differences between seven morphs of Icelandic Arctic charr (FJ, Fljótaá; VS, Vatnshlíðarvatn silver; VB, Vatnshlíðarvatn brown; SV, Svínavatn; TP, Þingvallavatn pelagic; TLB, Þingvallavatn large benthic; and GB, Galtaból benthic) in: (a) size at hatching (H) and (b) its relationship with egg size; and (c) size at first feeding (FF) and (d) its relationship with egg size. Morphs from the same lake share similar colors and are ordered according to phenotypic proximity to the ancestral anadromous morph (FJ). Letters indicate significant differences (*p* < .05; a, c). For relationships between size at H or FF and egg size (b, d), significant pairwise comparisons of slopes are showed in legend using lines and associated significance level, whereas slopes that differed from zero are shown in legend using significance levels only (*p* < .05*; *p* < .01**; *p* < .001***; *p* < .0001). Shaded areas represent 95% confidence intervals and morphs indicated by different symbols and colors.

Morphs differed in size at H (*F*_6,85_ = 15.29, *p* < .0001), with TP embryos hatching larger (16.7 mm) than those of all other morphs, and TLB embryos hatching at a larger size (15.7 mm) compared to VS/VB (14.5 mm for both; all Tukey's pairwise: *p* < .05; Figure 6a). There was no effect of egg size on size at H (Figure 6b). Morphs differed in size at FF (*F*_6,90_ = 33.37, *p* < .0001) with most pairwise comparisons being significant (Tukey's pairwise: *p* < .05), except for: (1) FJ (LSM = 20.3 mm), TLB (20.4 mm), and GB (20 mm); (2) TP (21.3 mm) and TLB; (3) VS and VB; and finally (4) TLB and GB (Figure 6c).

Within all morphs, larger eggs resulted in larger size at FF (Figure 6d). However, the significant interaction between morph × egg size indicates that the effect of egg size on size at FF varied among morphs (*F*_6,83_ = 2.93, *p =* .012; Table 4). Specifically, in FJ, VB, SV, and GB egg size was more strongly correlated with size at FF than in the three other morphs (slopes: *p <* .01, Table 4, Figure 6d). Finally, the pairwise differences between the slopes further showed that for a given egg size, FF offspring from the GB morph were larger than FF offspring in the VS and VB morphs (*F*_1,83_ = 23.23, *p* < .0001; Figure 6c,d).

## 4 DISCUSSION

Developmental (phenotypic) plasticity has been proposed to affect evolution by facilitating adaptive change (West‐Eberhard, 2003), yet the role that egg size may play in the diversification of natural populations is only beginning to be understood (Beck et al., 2019, 2020; Cogliati et al., 2018; Leblanc et al., 2011, 2016; Penney et al., 2018; Pfennig & Martin, 2009, 2010; Smalås et al., 2017). This study characterized egg size and development among seven morphs of Arctic charr. The effects of egg size on offspring traits were most prominent at first feeding (FF), whereby larger eggs produced larger offspring. However, egg size had no effect on developmental timings. Although there were morph differences in egg size and developmental timings, there is very little evidence to suggest that these differences are due to the extent of phenotypic divergence from the ancestral anadromous morph. Differences in early life‐history traits can have large impacts on offspring fitness (Hutchings, 1991; Krist, 2011) and we, therefore, discuss how egg size variation may interact with the environment to influence the development and/or maintenance of the morphs included in this study.

### 4.1 Female phenotype

Divergence in age and size at maturity are among the life‐history characteristics that are associated with the occurrence of multiple sympatric morphs (Klemetsen, 2010; Sandlund et al., 1992; Skoglund et al., 2015), as evidenced by the older and larger females from TLB compared to other morphs in this study (Figure 2). Such increases in size and age generally have a positive relationship with lifetime reproductive success (see review by Koch & Narum, 2021) and may be indicative of repeated spawning events (i.e., iteroparity) throughout an individual's lifetime. However, repeated spawning may come at a cost of reduced reproductive success due to the life‐history trade‐off between energy investment in current or future reproductive events (Christie et al., 2018; Seamons & Quinn, 2010), which depends upon an individual's survival. Variation in energy investment between each breeding season in iteroparous individuals is also likely to have a consequence on egg size, yet there are few, if any, studies examining egg size differences among successive breeding seasons within an iteroparous population. Furthermore, the extent to which repeated spawning events might contribute to phenotypic variance and subsequent divergence has also yet to be explored.

### 4.2 Egg size patterns across morphs

Egg size differences between Arctic charr morphs have been widely reported and range between 3.2 mm and 6.1 mm in diameter (Baroudy & Elliott, 1994; Pavlov & Osinov, 2008; Sandlund et al., 1992; Smalås et al., 2017; Sparholt, 1985). We found egg sizes in Icelandic Arctic charr morphs to be within this range (3.72–5.64 mm). The anadromous FJ and two morphs from Vatnshlíðarvatn (VS/VB) had the smallest egg sizes (Figure 3), although the small egg size in VB is likely due to the small size of mothers (Figure S2). Both FJ and VS share a migratory reproductive strategy (although with different distances), spawning in small streams, and incubate their eggs in riverine environments. Migratory salmonids that face increased water velocities (Braun et al., 2013) and/or experience longer distances to spawning grounds (Fleming & Gross, 1989; Kinnison et al., 2001) tend to produce smaller eggs, suggesting that environmental conditions favor smaller size in more fluvial environments, or that energy spent by migrating females is at the expense of reduced reproductive investment per egg (Braun et al., 2013). The timing of oogenesis in Arctic charr varies by locality (Kuznetsov & Mosyagina, 2016), with egg size variation potentially reflecting maternal food availability and/or temperature differences between habitats during maturation, in addition to genotype. Precise timing and duration of when females migrate to spawning grounds for the morphs studied here are unknown.

Temperature differences between spawning habitats were evident by the larger eggs in SV, which were concordant with higher temperatures observed at the fishing site (which ranged from 11°C to 7°C between September and November, when they spawned). Such large eggs observed in this morph may be constrained by oxygen due to warmer temperature (Einum & Fleming, 2002). However, it is still unclear if mature fish from SV incubate their eggs at the precise location where they were caught. Although water temperature is potentially a factor shaping egg size and egg number in Arctic charr, interpreting our results in the context of egg size evolution in response to temperature would be speculative with the current study design. This is not unique to our study, and in fact very little is known about the natural thermal conditions for egg incubation (i.e., from spawning to emergence) in many species of salmonids and/or in divergent populations. Moreover, the environment experienced by the mother during oogenesis and oocyte maturation is very rarely characterized, apart from reports of striking migratory distances in salmonids (e.g., Quinn & Myers, 2004; Strøm et al., 2018). Thus, the complex response of egg size to natural selection in wild populations of salmonids remains an evolutionary puzzle. Because ecological factors are important determinants of egg size and fitness of juvenile salmonids (e.g., Cogliati et al., 2018; Jonsson & Greenberg, 2022; Self et al., 2018), further work is needed to better characterize the environment both during maturation and during embryonic development.

We hypothesized that there will be a decrease in egg size variation as morphs become more phenotypically diverged from the ancestral anadromous morph (FJ), yet only the pelagic morph from Þingvallavatn (TP) had significantly less variation in egg size FJ (Figure 3d). Reduced variation in egg size may be reflective of lower levels of phenotypic diversity, including plasticity, as individuals become more specialized on alternative resources. Indeed, when reared under common‐garden conditions, discrete sympatric morphs lake þingvallavatn (TP/TLB) showed less morphological plasticity in response to diet than the more subtly diverged sympatric morphs from lake Vatnshlíðarvatn (VS/VB; Parsons et al., 2010, 2011). As morphs become more attuned to their environment, the need for plasticity in developmental processes may also be reduced (Waddington, 1959; West‐Eberhard, 2003), since plasticity can be costly (DeWitt et al., 1998). In contrast, the high variation in egg size for the anadromous FJ may be reflective of a bet hedging strategy to unpredictable environmental conditions in spawning streams (Koops et al., 2003; Moir et al., 2002; Slatkin, 1974; Steel et al., 2012). Changes in egg size, as well as changes in thermal regimes, can alter developmental time and directly influence survival by causing mismatches between development (e.g., emergence time or time to reach later life‐history stages) and the environment (e.g., flow, predation and food) (Crozier et al., 2008; Isaak et al., 2012; Steel et al., 2012). Differences in the maternally endowed resource environment not only reflect adaptive maternal effects, such as investment in larger eggs in poorer environments (Braun et al., 2013), but may also increase the potential for plasticity in how offspring utilize resources during development (e.g., Landberg, 2014; Pfennig & Martin, 2009).

### 4.3 Variation in offspring phenotype among morphs

Morphs differed extensively in their rate of development with differences existing in time to hatching between VS and VB (with VS hatching earlier than VB), but not between TP and TLB. Offspring from FJ hatch earlier than all other morphs, suggesting that earlier hatching may be an adaptation to stream habitats with riskier environments (e.g., increased predation risk; Mirza et al., 2001). Although Arctic charr from FJ had small eggs, there was no effect of egg size on hatching time.

Despite having smaller egg size and hatching earlier, FJ offspring were larger at FF and took longer to develop to FF compared to VS and VS/VB, respectively. Jonsson and Jonsson (2021) showed that offspring from anadromous brown trout (*Salmo trutta*) parents grew faster than offspring from resident parents of the same river, suggesting that better growth was due to differences in gene expression. Indeed, studies on gene expression in offspring from FJ show high correlation of *Mmp9* (a growth‐related gene involved in the development of the feeding apparatus; Sharif et al., 2014) with offspring size at hatching (Beck S. V., Räsänen K., Kristjánsson B. K., Leblanc C. A., Unpublished). Such compensatory growth has also been seen in a mouthbrooding cichlid (*Simochromis pleurospilus*), whereby egg size‐dependent expression of a growth‐related gene (the growth hormone receptor, *GHR*) enabled faster growth in offspring from smaller eggs (Segers et al., 2012). Our combined results (here and the currently unpublished gene expression study) reveal that differences in developmental rate in Arctic charr are underlined by differential gene expression that may be mediated through egg size.

FF is considered a critical development stage (May, 1974) where offspring begin feeding and have a specific window of opportunity to learn how to feed. We found that larger offspring came from larger eggs by the onset of FF in all morphs except those from þingvallavatn and the VS morph (Figure 6d). Differences in size at FF can have considerable implications for survival (Boubee & Ward, 1997; Dial et al., 2017) and the availability of possible diet items. In cod (*Gadus morhua*) for instance, gape size of larvae at FF is strongly positively correlated with egg size (Knutsen & Tilseth, 1985). Egg size‐mediated changes in feeding behavior (Leblanc et al., 2011) combined with size‐correlated constraints on diet choice (mediated by offspring size and associated mouth gape), can ultimately promote divergence in alternative resource use in the wild, especially in organisms with highly plastic trophic morphologies (Adams & Huntingford, 2004; Parsons et al., 2016; Robinson & Wilson, 1994). Trophic specializations can occur early in development in Arctic charr, as documented between morphs (e.g., þingvallavatn; Kapralova et al., 2015) and even between families (e.g., Vatnshlíðarvatn, Beck et al., 2020). However, even though salmonids are widely studied in evolutionary biology to understand the process of diversification, very little is known about variation between sympatric morphs or populations during early life stages and their ecology (both habitat and feeding) in the wild.

## 5 CONCLUSION

Further studies on the drivers of variation in maternal investment patterns should include variation in fecundity to infer variation in optimal strategies (e.g., per propagule investment and trade‐offs between egg size and egg number) of different resource morphs. Furthermore, studies comparing early developmental traits in morphs that vary in their degree of phenotypic and genetic divergence are needed to identify and disentangle mechanisms at play in shaping diversity of rapidly evolving species. Along with the characterization of the ecology of egg incubation in the wild, these studies would increase our chances of understanding and conserving the diversity of salmonids. Our findings provide a foundation for future work by characterizing egg size across several morphs and highlighting how such fine‐scale variation in developmental processes may initiate and/or maintain phenotypic diversity in polymorphic systems.

## AUTHOR CONTRIBUTIONS

**Samantha V. Beck:** Conceptualization (equal); data curation (lead); formal analysis (lead); funding acquisition (supporting); investigation (lead); methodology (lead); validation (supporting); visualization (lead); writing – original draft (lead); writing – review and editing (lead). **Katja Räsänen:** Conceptualization (equal); formal analysis (supporting); funding acquisition (supporting); methodology (supporting); supervision (supporting); validation (supporting); writing – original draft (supporting); writing – review and editing (supporting). **Bjarni K. Kristjánsson:** Conceptualization (equal); funding acquisition (equal); investigation (supporting); methodology (supporting); project administration (supporting); resources (equal); supervision (supporting); validation (supporting); writing – original draft (supporting); writing – review and editing (supporting). **Skúli Skúlason:** Conceptualization (equal); funding acquisition (supporting); project administration (supporting); resources (supporting); supervision (supporting); writing – original draft (supporting). **Zophonías O. Jónsson:** Conceptualization (supporting); funding acquisition (supporting); project administration (supporting); supervision (equal); writing – original draft (supporting); writing – review and editing (supporting). **Markos Tsinganis:** Formal analysis (supporting); investigation (supporting); methodology (supporting). **Camille A. Leblanc:** Conceptualization (equal); data curation (supporting); formal analysis (supporting); funding acquisition (lead); investigation (supporting); methodology (supporting); project administration (lead); resources (supporting); supervision (lead); validation (lead); visualization (supporting); writing – original draft (supporting); writing – review and editing (supporting).

## CONFLICT OF INTEREST

There are no conflicts of interest.

## Supporting information


Appendix S1

## Data Availability

Datasets used in this study can be accessed at https://doi.org/10.5061/dryad.jh9w0vtfg and R scripts are available at https://github.com/SamVBeck/egg_size_Icelandic_Arctic_charr.
