# Supplementary material for: Variation in egg size and offspring phenotype among and within seven Arctic charr morphs
Source: Ecol Evol. 2022 Oct 18;12(10):e9427. doi: 10.1002/ece3.9427 (PMC9577412; doi:10.1002/ece3.9427)
Supplement: Supplementary file 1 — Appendix S1 [file ECE3-12-e9427-s001.docx]

**Appendix**

**Figure S1** Morph differences in female fork length (FL,cm) in seven morphs of Icelandic Arctic charr (FJ, Fljótaá; VS, Vatnshlíðarvatn silver; VB, Vatnshlíðarvatn brown; SV, Svínavatn; TP, Þingvallavatn pelagic; TLB, Þingvallavatn large benthic; and GB, Galtaból benthic), indicated by different colours. Sympatric morphs share similar colours and are ordered according to phenotypic proximity to the ancestral anadromous morph (FJ). Letters indicate significant differences (P <0.05).

**Figure S2** Relationship between female size (fork length, cm) and egg size (mm) in seven morphs of Icelandic Arctic charr (FJ, Fljótaá; VS, Vatnshlíðarvatn silver; VB, Vatnshlíðarvatn brown; SV, Svínavatn; TP, Þingvallavatn pelagic; TLB, Þingvallavatn large benthic; and GB, Galtaból benthic). Different colours and symbols represent each morph, which are ordered according to phenotypic proximity to the ancestral anadromous morph (FJ). Sympatric morphs share similar colours. Shaded areas represent 95% confidence intervals.

| **Table S1** Sampling design, measurements of female and offspring traits and developmental timing in seven morphs of Arctic charr (*Salvelinus alpinus*) found in allopatry and sympatry throughout Iceland. The seven populations/morphs are FJ, Fljótaá (ancestral anadromous population); VS, Vatnshlíðarvatn silver; VB, Vatnshlíðarvatn brown; SV, Svínavatn; TLB, Þingvallavatn large benthic; TP, Þingvallavatn planktivorous; and GB, Galtaból benthic. | | | | | | | | | | | | | | | | | | | | |  |
| --- | --- | --- | --- | --- | --- | --- | --- | --- | --- | --- | --- | --- | --- | --- | --- | --- | --- | --- | --- | --- | --- |
|  |  |  |  |  |  |  |  |  |  |  |  |  |  |  |  |  |  |  |  |  |  |
|  |  |  |  |  |  |  |  |  |  |  |  |  |  |  |  |  |  |  |  |  |  |
| **Morph** | **♀ ID** | **♀ age**  **(years)** | **♀ FL**  **(cm)** | **♂ ID** | **Egg size** | | | | | | **Hatching** | | | | | **First feeding** | | | | |  |
|  |  |  |  |  | **N** | **#cages** | **Mean egg size (mm)** | **CV (%)** | **PF DD** | **E DD** | **N** | **#cages** | **Mean SL (mm)** | **CV (%)** | **DD** | **N** | **# of cages** | **Mean SL (mm)** | **CV (%)** | **DD** |  |
| **FJ** | **292** | **–** | **33.3** | **297** | **15** | **1** | **4.70 ± 0.28** | **6.1** | **9** | **205** | **18** | **1** | **14.19 ± 0.36** | **2.54** | **388** | **10** | **1** | **21.35 ± 0.52** | **2.45** | **668** |  |
| **FJ** | **294** | **7** | **39.4** | **296** | **148** | **8** | **4.56 ± 0.15** | **3.3** | **9** | **205** | **80** | **4** | **13.81 ± 0.55** | **4.01** | **388** | **22** | **1** | **20.86 ± 0.72** | **3.46** | **707** |  |
| **FJ** | **295** | **6** | **35.8** | **297** | **17** | **1** | **4.40 ± 0.13** | **3** | **9** | **205** | **12** | **1** | **15.14 ± 0.43** | **2.84** | **417** | **11** | **1** | **20.29 ± 0.83** | **4.11** | **638** |  |
| **FJ** | **298** | **6** | **37.5** | **300** | **97** | **5** | **4.82 ± 0.21** | **4.3** | **9** | **201** | **65** | **3** | **13.83 ± 0.62** | **4.47** | **381** | **24** | **1** | **20.90 ± 0.91** | **4.35** | **707** |  |
| **FJ** | **299** | **4** | **31.5** | **300** | **65** | **2** | **4.14 ± 0.31** | **7.5** | **9** | **201** | **20** | **1** | **13.25 ± 0.58** | **4.38** | **405** | **10** | **1** | **19.10 ± 0.88** | **4.62** | **676** |  |
| **FJ** | **308** | **3** | **35.6** | **318** | **51** | **2** | **4.53 ± 0.40** | **8.8** | **10** | **199** | **20** | **2** | **15.71 ± 0.89** | **5.68** | **443** | **41** | **2** | **20.56 ± 0.78** | **3.77** | **649** |  |
| **FJ** | **309** | **4** | **35.8** | **316** | **53** | **3** | **4.58 ± 0.17** | **3.7** | **10** | **199** | **18** | **3** | **15.82 ± 0.64** | **4.04** | **441** | **20** | **1** | **20.12 ± 0.63** | **3.13** | **649** |  |
| **FJ** | **311** | **4** | **46** | **317** | **52** | **3** | **4.80 ± 0.12** | **2.4** | **10** | **199** | **18** | **3** | **16.54 ± 0.36** | **2.16** | **441** | **41** | **2** | **20.93 ± 0.53** | **2.51** | **649** |  |
| **FJ** | **312** | **4** | **40.2** | **318** | **46** | **4** | **4.32 ± 0.12** | **2.8** | **10** | **204** | **48** | **8** | **15.12 ± 0.43** | **2.85** | **444** | **41** | **2** | **19.18 ± 0.68** | **3.53** | **649** |  |
| **FJ** | **313** | **3** | **37.8** | **318** | **35** | **3** | **4.34 ± 0.11** | **2.5** | **10** | **204** | **24** | **4** | **15.45 ± 0.44** | **2.88** | **435** | **20** | **1** | **19.23 ± 0.90** | **4.69** | **649** |  |
| **FJ** | **314** | **4** | **41.5** | **317** | **100** | **6** | **4.71 ± 0.12** | **2.5** | **10** | **199** | **36** | **6** | **15.70 ± 0.53** | **3.37** | **446** | **20** | **1** | **20.79 ± 0.47** | **2.28** | **649** |  |
| **FJ** | **315** | **4** | **32.5** | **316** | **24** | **1** | **4.38 ± 0.17** | **3.9** | **10** | **204** | **20** | **2** | **15.55 ± 0.47** | **2.99** | **441** | **20** | **1** | **20.90 ± 0.73** | **3.48** | **649** |  |
| **FJ** | **338** | **3** | **37.3** | **344** | **19** | **2** | **4.38 ± 0.09** | **2.2** | **10** | **198** | **5** | **1** | **16.52 ± 0.50** | **3.01** | **443** | **14** | **1** | **20.63 ± 0.52** | **2.54** | **612** |  |
| **FJ** | **339** | **5** | **32.4** | **345** | **38** | **2** | **4.52 ± 0.21** | **4.6** | **10** | **198** | **20** | **2** | **15.85 ± 0.74** | **4.66** | **433** | **45** | **2** | **19.51 ± 0.69** | **3.52** | **617** |  |
| **FJ** | **343** | **3** | **35.5** | **345** | **50** | **4** | **4.37 ± 0.06** | **1.3** | **10** | **198** | **25** | **5** | **14.83 ± 0.70** | **4.7** | **422** | **20** | **1** | **19.41 ± 0.50** | **2.55** | **612** |  |
| GB | 38 | 5 | 19.9 | 51 | 18 | 2 | 4.99 ± 0.15 | 3 | 11 | 230 |  |  |  |  |  | 10 | 2 | 20.67 ± 0.51 | 2.44 | 618 |  |
| GB | 39 | 5 | 21.1 | 51 | 18 | 2 | 5.01 ± 0.18 | 3.7 | 11 | 230 |  |  |  |  |  |  |  |  |  |  |  |
| GB | 40 | 5 | 22.3 | 52 | 18 | 2 | 5.14 ± 0.12 | 2.3 | 11 | 230 |  |  |  |  |  | 10 | 2 | 20.85 ± 0.49 | 2.33 | 618 |  |
| GB | 41 | 5 | 22.6 | 53 | 27 | 3 | 5.19 ± 0.10 | 1.9 | 11 | 230 |  |  |  |  |  |  |  |  |  |  |  |
| GB | 42 | 7 | 22.3 | 50 | 27 | 3 | 5.36 ± 0.13 | 2.4 | 11 | 230 |  |  |  |  |  | 9 | 3 | 21.69 ± 1.18 | 5.46 | 618 |  |
| GB | 43 | 5 | 19.4 | 49 | 27 | 3 | 5.21 ± 0.10 | 1.8 | 11 | 230 |  |  |  |  |  | 9 | 3 | 20.45 ± 0.59 | 2.88 | 618 |  |
| GB | 44 | 5 | 21.5 | 52 | 27 | 3 | 5.12 ± 0.15 | 2.9 | 11 | 230 |  |  |  |  |  | 10 | 3 | 19.82 ± 0.77 | 3.88 | 618 |  |
| GB | 45 | 6 | 21.7 | 50 | 27 | 3 | 5.19 ± 0.15 | 2.8 | 11 | 230 |  |  |  |  |  | 9 | 3 | 20.78 ± 0.60 | 2.86 | 618 |  |
| GB | 46 | 7 | 23.6 | 51 | 27 | 3 | 5.32 ± 0.10 | 2 | 11 | 230 |  |  |  |  |  | 9 | 3 | 22.2 ± 0.48 | 2.18 | 618 |  |
| GB | 47 | 8 | 24.6 | 50 | 18 | 3 | 5.35 ± 0.18 | 3.4 | 11 | 230 |  |  |  |  |  |  |  |  |  |  |  |
| GB | 48 | 6 | 20.5 | 49 | 27 | 3 | 5.07 ± 0.10 | 1.9 | 11 | 230 |  |  |  |  |  | 10 | 2 | 20.78 ± 0.43 | 2.05 | 618 |  |
| GB | 164 | 6 | 25.6 | 170 | 24 | 2 | 5.11 ± 0.14 | 2.7 | 10 | 226 | 26 | 2 | 15.38 ± 0.39 | 2.57 | 438 | 12 | 1 | 18.41 ± 0.72 | 3.93 | 619 |  |
| GB | 166 | 6 | 21.2 | 171 | 15 | 1 | 4.73 ± 0.10 | 2 | 10 | 226 | 17 | 1 | 14.84 ± 0.58 | 3.89 | 450 | 14 | 1 | 17.97 ± 1.63 | 9.05 | 619 |  |
| GB | 167 | 6 | 20.7 | 169 | 15 | 1 | 5.08 ± 0.18 | 3.5 | 10 | 226 | 18 | 1 | 14.31 ± 0.67 | 4.67 | 425 | 12 | 1 | 17.97 ± 0.60 | 3.36 | 619 |  |
| GB | 168 | 6 | 18.7 | 170 | 19 | 1 | 4.79 ± 0.16 | 3.3 | 10 | 226 | 13 | 1 | 14.05 ± 0.42 | 2.97 | 450 | 10 | 1 | 18.30 ± 0.30 | 1.66 | 619 |  |
| SV | 172 | 5 | 28.8 | 174 | 20 | 2 | 5.64 ± 0.29 | 5.1 | 10 |  | 19 | 1 | 16.31 ± 0.48 | 2.97 | 454 |  |  |  |  |  |  |
| SV | 173 | 6 | 24.3 | 174 | 17 | 2 | 5.09 ± 0.08 | 1.6 | 10 |  | 11 | 1 | 16.69 ± 0.73 | 4.36 | 411 |  |  |  |  |  |  |
| SV | 200 | 6 | 24.3 | 203 | 21 | 2 | 5.15 ± 0.16 | 3.2 | 9 |  | 16 | 1 | 14.69 ± 0.67 | 4.54 | 403 |  |  |  |  |  |  |
| SV | 202 | 4 | 24.2 | 203 | 19 | 1 | 5.61 ± 0.34 | 6.1 | 9 |  | 17 | 1 | 14.29 ± 0.41 | 2.85 | 399 |  |  |  |  |  |  |
| SV | 204 | 6 | 26.2 | 209 | 22 | 1 | 5.50 ± 0.23 | 4.1 | 9 | 203 |  |  |  |  |  | 11 | 1 | 22.54 ± 0.51 | 2.26 | 718 |  |
| SV | 205 | 5 | 27 | 208 | 22 | 1 | 5.56 ± 0.19 | 3.3 | 9 | 203 |  |  |  |  | 426 | 7 | 1 | 25.35 ± 0.70 | 2.74 | 697 |  |
| SV | 206 | 9 | 26.5 | 208 | 26 | 1 | 5.45 ± 0.17 | 3.2 | 9 | 203 |  |  |  |  | 407 | 10 | 1 | 22.28 ± 0.67 | 3.01 | 766 |  |
| SV | 207 | 6 | 25.5 | 209 | 26 | 1 | 5.16 ± 0.20 | 3.9 | 9 | 203 |  |  |  |  | 394 | 10 | 1 | 21.7 ± 0.52 | 2.4 | 718 |  |
| SV | 248 | 4 | 25.3 | 249 | 20 | 1 | 5.04 ± 0.12 | 2.4 | 10 | 202 |  |  |  |  |  |  |  |  |  |  |  |
| SV | 250 | 4 | 24.9 | 259 | 22 | 1 | 4.95 ± 0.20 | 4 | 10 |  |  |  |  |  |  |  |  |  |  |  |  |
| SV | 251 | 5 | 25.7 | 258 | 22 | 1 | 5.32 ± 0.12 | 2.3 | 10 |  |  |  |  |  |  |  |  |  |  |  |  |
| SV | 252 | 5 | 25.9 | 259 | 22 | 1 | 5.45 ± 0.15 | 2.8 | 10 |  |  |  |  |  |  |  |  |  |  |  |  |
| SV | 253 | 4 | 25.1 | 259 | 24 | 1 | 5.52 ± 0.23 | 4.1 | 10 |  |  |  |  |  |  |  |  |  |  |  |  |
| SV | 254 | 5 | 26.4 | 260 | 20 | 1 | 5.54 ± 0.26 | 4.6 | 10 | 202 |  |  |  |  |  |  |  |  |  |  |  |
| SV | 255 | 4 | 22.4 | 258 | 22 | 1 | 5.05 ± 0.16 | 3.2 | 10 |  |  |  |  |  |  |  |  |  |  |  |  |
| SV | 256 | 4 | 28.5 | 260 | 22 | 1 | 5.32 ± 0.21 | 4 | 10 | 202 |  |  |  |  |  |  |  |  |  |  |  |
| SV | 257 | 4 | 24.3 | 260 | 20 | 1 | 5.20 ± 0.25 | 4.9 | 10 | 206 |  |  |  |  |  |  |  |  |  |  |  |
| **TLB** | **1** | **9** | **37.1** | **13** | **64** | **4** | **5.26 ± 0.24** | **4.5** | **14** | **207** | **7** | **3** | **17.15 ± 0.35** | **2.05** | **442** | **18** | **3** | **21.13 ± 0.67** | **3.17** | **680** |  |
| **TLB** | **2** | **8** | **30** | **12** | **59** | **3** | **5.11 ± 0.20** | **3.8** | **16** | **207** | **10** | **2** | **16.28 ± 0.43** | **2.65** | **443** | **16** | **2** | **20.40 ± 0.71** | **3.48** | **680** |  |
| **TLB** | **3** | **6** | **35.2** | **14** | **57** | **4** | **4.84 ± 0.20** | **4.1** | **14** | **207** | **15** | **5** | **15.88 ± 1.29** | **8.12** | **450** | **28** | **5** | **20.89 ± 1.04** | **4.95** | **677** |  |
| **TLB** | **5** | **9** | **37.8** | **15** | **61** | **4** | **5.08 ± 0.23** | **4.5** | **14** | **207** |  |  |  |  |  | **30** | **5** | **20.71 ± 0.42** | **2.01** | **670** |  |
| **TLB** | **6** | **10** | **36.1** | **14** | **58** | **4** | **5.02 ± 0.19** | **3.8** | **15** | **207** | **15** | **5** | **16.19 ± 0.70** | **4.33** | **421** | **30** | **5** | **20.49 ± 0.58** | **2.83** | **672** |  |
| **TLB** | **7** | **9** | **32.3** | **15** | **56** | **3** | **5.06 ± 0.17** | **3.3** | **16** | **207** |  |  |  |  |  | **28** | **3** | **21.20 ± 0.71** | **3.33** | **650** |  |
| **TLB** | **8** | **11** | **41.1** | **14** | **53** | **4** | **4.83 ± 0.21** | **4.4** | **14** | **207** | **6** | **2** | **16.47 ± 0.68** | **4.16** | **433** | **24** | **4** | **20.64 ± 0.84** | **4.06** | **677** |  |
| **TLB** | **9** | **7** | **29.7** | **12** | **57** | **3** | **5.14 ± 0.20** | **3.9** | **16** | **207** | **10** | **2** | **16.55 ± 0.24** | **1.44** | **441** | **17** | **3** | **20.41 ± 1.43** | **6.98** | **672** |  |
| **TLB** | **10** | **11** | **28.5** | **12** | **46** | **3** | **5.03 ± 0.14** | **2.8** | **16** | **207** | **8** | **1** | **16.31 ± 0.90** | **5.52** | **424** | **22** | **3** | **20.73 ± 0.62** | **3** | **665** |  |
| **TLB** | **11** | **11** | **37** | **15** | **64** | **4** | **5.07 ± 0.18** | **3.6** | **14** | **207** | **6** | **2** | **16.72 ± 0.34** | **2.01** | **436** | **12** | **2** | **21.42 ± 0.68** | **3.16** | **656** |  |
| TLB | 112 | 9 | 36.1 | 134 | 41 | 1 | 5.42 ± 0.15 | 2.8 | 12 | 220 | 10 | 2 | 12.92 ± 0.73 | 5.65 | 446 |  |  |  |  |  |  |
| TLB | 115 | 15 | 49.9 | 134 | 18 | 1 | 5.00 ± 0.28 | 5.7 | 12 | 220 | 30 | 5 | 13.87 ± 1.04 | 7.53 | 434 |  |  |  |  |  |  |
| **TLB** | **116** | **8** | **40.3** | **132** | **59** | **2** | **5.17 ± 0.25** | **4.8** | **12** | **220** | **15** | **3** | **14.56 ± 1.52** | **10.43** | **445** | **15** | **1** | **19.51 ± 0.76** | **3.92** | **643** |  |
| TLB | 117 | 8 | 42.8 | 131 | 19 | 1 | 5.06 ± 0.24 | 4.7 | 12 | 220 |  |  |  |  |  |  |  |  |  |  |  |
| **TLB** | **119** | **7** | **39.1** | **134** | **17** | **1** | **4.87 ± 0.14** | **2.8** | **12** | **220** | **16** | **2** | **15.91 ± 0.97** | **6.08** | **425** | **14** | **1** | **18.69 ± 0.77** | **4.14** | **643** |  |
| **TLB** | **124** | **7** | **35.9** | **130** | **41** | **1** | **5.10 ± 0.18** | **3.5** | **12** | **220** | **10** | **2** | **15.43 ± 0.58** | **3.77** | **433** | **10** | **1** | **20.16 ± 0.73** | **3.64** | **643** |  |
| **TLB** | **128** | **8** | **40** | **129** | **18** | **1** | **4.58 ± 0.31** | **6.7** | **12** | **220** | **11** | **2** | **15.22 ± 0.62** | **4.1** | **432** | **16** | **1** | **19.42 ± 2.17** | **11.16** | **660** |  |
| TP | 82 | 7 | 21 | 104 | 18 | 3 | 4.73 ± 0.21 | 4.5 | 13 | 226 | 9 | 3 | 16.05 ± 0.85 | 5.33 | 442 | 9 | 3 | 21.84 ± 1.04 | 4.75 | 654 |  |
| **TP** | **83** | **6** | **21** | **111** | **41** | **4** | **5.12 ± 0.25** | **4.9** | **14** | **226** | **9** | **3** | **15.94 ± 2.09** | **13.09** | **461** | **9** | **3** | **21.30 ± 0.76** | **3.59** | **634** |  |
| **TP** | **84** | **7** | **19.5** | **103** | **29** | **4** | **5.11 ± 0.45** | **8.9** | **12** | **226** | **9** | **3** | **16.64 ± 0.80** | **4.78** | **453** | **9** | **3** | **22.14 ± 0.82** | **3.69** | **642** |  |
| **TP** | **85** | **5** | **19.2** | **105** | **23** | **4** | **5.08 ± 0.23** | **4.5** | **16** | **226** | **9** | **3** | **17.30 ± 0.62** | **3.56** | **457** | **9** | **3** | **22.26 ± 0.61** | **2.73** | **638** |  |
| **TP** | **86** | **7** | **20.4** | **104** | **29** | **4** | **4.88 ± 0.20** | **4** | **14** | **226** | **9** | **3** | **17.32 ± 0.64** | **3.67** | **457** | **9** | **3** | **22.16 ± 0.60** | **2.7** | **642** |  |
| TP | 88 | 6 | 19.6 | 102 | 15 | 3 | 4.91 ± 0.21 | 4.3 | 16 | 226 | 9 | 3 | 16.44 ± 0.49 | 2.96 | 453 | 9 | 3 | 21.82 ± 0.59 | 2.71 | 654 |  |
| TP | 89 | 7 | 20.1 | 102 | 15 | 3 | 5.05 ± 0.32 | 6.4 | 14 | 226 | 6 | 2 | 17.12 ± 0.48 | 2.81 | 453 | 6 | 2 | 22.04 ± 0.87 | 3.96 | 654 |  |
| TP | 91 | 5 | 20 | 105 | 18 | 3 | 4.96 ± 0.10 | 1.9 | 14 | 226 | 11 | 3 | 17.19 ± 1.26 | 7.33 | 461 | 9 | 3 | 22.07 ± 1.27 | 5.76 | 654 |  |
| **TP** | **92** | **5** | **20.1** | **105** | **28** | **4** | **4.92 ± 0.24** | **4.9** | **14** | **226** | **9** | **3** | **16.92 ± 0.83** | **4.9** | **453** | **9** | **3** | **22.10 ± 1.05** | **4.74** | **636** |  |
| TP | 99 | 4 | 19.6 | 103 | 23 | 4 | 4.90 ± 0.24 | 5 | 16 | 226 | 9 | 3 | 16.08 ± 0.46 | 2.86 | 453 | 10 | 2 | 21.15 ± 0.90 | 4.24 | 654 |  |
| **TP** | **240** | **5** | **21.2** | **239** | **16** | **1** | **5.09 ± 0.11** | **2.2** | **9** | **263** | **13** | **1** | **16.23 ± 0.44** | **2.69** | **408** | **14** | **1** | **20.97 ± 1.00** | **4.78** | **681** |  |
| TP | 241 | 6 | 20.6 | 239 | 16 | 1 | 4.96 ± 0.18 | 3.7 | 9 | 263 | 15 | 1 | 16.26 ± 0.69 | 4.24 | 431 |  |  |  |  |  |  |
| **TP** | **250** | **5** | **22.5** | **245** | **22** | **2** | **4.88 ± 0.31** | **6.3** | **9** | **263** | **6** | **1** | **15.61 ± 0.87** | **5.56** | **448** | **11** | **1** | **20.87 ± 0.71** | **3.39** | **672** |  |
| **TP** | **251** | **5** | **21.2** | **249** | **16** | **1** | **5.27 ± 0.22** | **4.1** | **9** | **263** | **8** | **1** | **17.18 ± 0.33** | **1.91** | **431** | **11** | **1** | **21.02 ± 0.96** | **4.58** | **672** |  |
| **TP** | **252** | **4** | **22.6** | **245** | **17** | **1** | **5.15 ± 0.18** | **3.4** | **9** | **263** | **14** | **1** | **15.44 ± 0.61** | **3.97** | **408** | **12** | **1** | **21.31 ± 0.88** | **4.13** | **719** |  |
| **TP** | **253** | **7** | **22** | **249** | **17** | **1** | **5.03 ± 0.11** | **2.2** | **9** | **263** | **12** | **1** | **16.39 ± 0.46** | **2.78** | **431** | **11** | **1** | **20.28 ± 0.68** | **3.37** | **672** |  |
| TP | 319 | 6 | 21 | 337 | 19 | 1 | 4.78 ± 0.19 | 3.9 | 5 | 200 | 20 | 1 | 17.13 ± 0.71 | 4.14 | 450 | 21 | 1 | 21.32 ± 0.86 | 4.04 | 630 |  |
| TP | 320 | 5 | 19.9 | 335 | 20 | 1 | 4.67 ± 0.21 | 4.5 | 5 | 200 | 16 | 1 | 16.39 ± 0.74 | 4.52 | 457 | 22 | 1 | 19.64 ± 1.03 | 5.26 | 630 |  |
| TP | 323 | 5 | 18.5 | 336 | 16 | 1 | 4.75 ± 0.35 | 7.4 | 5 | 200 | 9 | 1 | 16.44 ± 0.53 | 3.22 | 457 | 21 | 1 | 20.38 ± 1.13 | 5.53 | 630 |  |
| TP | 324 | 7 | 23.7 | 334 | 15 | 1 | 5.25 ± 0.15 | 2.9 | 5 | 200 | 16 | 1 | 17.96 ± 0.64 | 3.56 | 453 | 20 | 1 | 22.22 ± 1.21 | 5.44 | 630 |  |
| TP | 325 | 6 | 19.7 | 337 | 15 | 1 | 4.87 ± 0.23 | 4.7 | 5 | 200 | 18 | 1 | 16.78 ± 0.58 | 3.44 | 453 | 22 | 1 | 20.89 ± 0.65 | 3.11 | 630 |  |
| TP | 329 | 6 | 20.2 | 335 | 17 | 1 | 4.89 ± 0.19 | 3.8 | 5 | 200 | 16 | 1 | 17.22 ± 0.67 | 3.92 | 453 | 20 | 1 | 21.08 ± 0.66 | 3.14 | 630 |  |
| TP | 330 | 5 | 22.9 | 333 | 18 | 1 | 4.84 ± 0.08 | 1.7 | 5 | 200 | 9 | 1 | 17.24 ± 0.91 | 5.26 | 457 | 20 | 1 | 20.84 ± 0.83 | 3.97 | 630 |  |
| TP | 332 | 7 | 21.3 | 333 | 20 | 1 | 4.88 ± 0.23 | 4.8 | 5 | 200 | 16 | 1 | 17.31 ± 0.43 | 2.49 | 446 | 20 | 1 | 20.36 ± 1.25 | 6.16 | 630 |  |
| **VB** | **16** | **5** | **17.6** | **34** | **48** | **4** | **4.61 ± 0.28** | **6.1** | **27** | **234** | **18** | **3** | **14.65 ± 1.21** | **8.24** | **464** | **18** | **3** | **19.04 ± 1.29** | **6.8** | **657** |  |
| **VB** | **17** | **7** | **17** | **34** | **32** | **3** | **4.87 ± 0.50** | **10.3** | **27** | **234** | **9** | **2** | **14.64 ± 1.13** | **7.75** | **466** | **20** | **2** | **18.81 ± 2.23** | **11.84** | **683** |  |
| **VB** | **18** | **5** | **14.5** | **36** | **31** | **3** | **3.77 ± 0.29** | **7.7** | **27** | **234** | **12** | **2** | **14.25 ± 1.40** | **9.84** | **456** | **15** | **2** | **18.05 ± 1.61** | **8.92** | **654** |  |
| VB | 19 | 8 | 18.9 | 36 | 17 | 4 | 4.71 ± 0.17 | 3.5 | 27 | 234 |  |  |  |  |  |  |  |  |  |  |  |
| **VB** | **23** | **5** | **16.3** | **32** | **19** | **3** | **3.72 ± 0.16** | **4.2** | **27** | **234** | **12** | **2** | **13.49 ± 0.61** | **4.53** | **462** | **20** | **2** | **16.04 ± 1.90** | **11.86** | **658** |  |
| **VB** | **24** | **6** | **15.2** | **33** | **32** | **3** | **3.78 ± 0.24** | **6.4** | **27** | **234** | **12** | **2** | **13.22 ± 0.37** | **2.78** | **462** | **20** | **2** | **17.56 ± 1.80** | **10.26** | **632** |  |
| **VB** | **25** | **6** | **18.6** | **35** | **38** | **4** | **4.83 ± 0.37** | **7.6** | **27** | **234** | **18** | **3** | **15.09 ± 1.08** | **7.17** | **460** | **18** | **3** | **17.65 ± 1.45** | **8.23** | **659** |  |
| **VB** | **26** | **4** | **13.7** | **32** | **32** | **3** | **3.72 ± 0.14** | **3.7** | **27** | **234** | **12** | **2** | **15.06 ± 1.93** | **12.78** | **458** | **17** | **2** | **15.86 ± 1.24** | **7.85** | **624** |  |
| **VB** | **28** | **8** | **20.6** | **35** | **47** | **4** | **5.36 ± 0.46** | **8.6** | **27** | **234** | **18** | **3** | **15.79 ± 1.09** | **6.9** | **463** | **18** | **3** | **18.44 ± 1.84** | **9.97** | **665** |  |
| **VB** | **30** | **4** | **14.7** | **32** | **22** | **3** | **4.31 ± 0.54** | **12.6** | **27** | **234** | **12** | **2** | **14.46 ± 1.42** | **9.79** | **464** | **19** | **2** | **17.14 ± 0.99** | **5.77** | **666** |  |
| **VB** | **31** | **4** | **14.2** | **35** | **41** | **4** | **4.01 ± 0.29** | **7.1** | **27** | **234** | **18** | **3** | **15.14 ± 1.14** | **7.5** | **458** | **18** | **3** | **16.48 ± 1.56** | **9.44** | **670** |  |
| VB | 135 | 5 | 17.6 | 160 | 19 | 2 | 4.51 ± 0.18 | 4 | 12 | 217 | 17 | 1 | 15.00 ± 0.76 | 5.05 | 470 | 25 | 1 | 18.14 ± 0.70 | 3.87 | 639 |  |
| VB | 143 | 6 | 24.8 | 156 | 72 | 3 | 4.59 ± 0.21 | 4.5 | 12 | 217 | 28 | 2 | 13.29 ± 0.68 | 5.12 | 386 | 24 | 1 | 18.11 ± 1.95 | 10.77 | 639 |  |
| VB | 148 | 7 | 26.4 | 161 | 17 | 2 | 4.49 ± 0.21 | 4.7 | 12 | 217 |  |  |  |  |  |  |  |  |  |  |  |
| VB | 149 | 7 | 17.2 | 163 | 53 | 2 | 4.36 ± 0.10 | 2.2 | 12 | 230 | 19 | 1 | 14.51 ± 0.87 | 6.02 | 441 | 9 | 1 | 17.53 ± 0.63 | 3.6 | 639 |  |
| VB | 150 | 8 | 24.1 | 158 | 24 | 2 | 5.10 ± 0.43 | 8.5 | 12 | 217 | 26 | 2 | 15.19 ± 1.07 | 7.02 | 429 | 25 | 1 | 19.66 ± 1.42 | 7.24 | 639 |  |
| VB | 152 | 5 | 15.7 | 161 | 96 | 2 | 4.31 ± 0.13 | 3 | 12 | 217 | 14 | 1 | 14.31 ± 0.51 | 3.55 | 442 | 17 | 1 | 17.38 ± 1.22 | 7.04 | 639 |  |
| **VS** | **54** | **5** | **23.3** | **73** | **22** | **3** | **4.57 ± 0.19** | **4.2** | **12** | **222** | **10** | **2** | **14.83 ± 1.43** | **9.63** | **433** | **13** | **2** | **19.17 ± 0.96** | **5.03** | **617** |  |
| **VS** | **56** | **6** | **24.5** | **73** | **27** | **3** | **4.18 ± 0.13** | **3.2** | **12** | **222** | **10** | **2** | **15.33 ± 0.70** | **4.57** | **435** | **11** | **1** | **18.5 ± 0.86** | **4.62** | **644** |  |
| **VS** | **65** | **7** | **27.8** | **74** | **49** | **4** | **4.69 ± 0.13** | **2.7** | **12** | **222** | **18** | **3** | **15.10 ± 0.86** | **5.67** | **435** | **18** | **3** | **18.92 ± 0.77** | **4.06** | **617** |  |
| **VS** | **67** | **5** | **21.7** | **74** | **36** | **3** | **4.30 ± 0.11** | **2.6** | **12** | **222** | **16** | **2** | **14.94 ± 0.74** | **4.98** | **431** | **20** | **2** | **18.21 ± 0.46** | **2.51** | **642** |  |
| **VS** | **68** | **7** | **22.5** | **72** | **33** | **4** | **4.61 ± 0.21** | **4.5** | **12** | **222** | **15** | **3** | **15.09 ± 0.60** | **3.98** | **427** | **15** | **2** | **19.19 ± 0.77** | **4** | **650** |  |
| **VS** | **69** | **5** | **22** | **73** | **32** | **3** | **4.21 ± 0.11** | **2.6** | **12** | **222** | **16** | **2** | **14.90 ± 0.55** | **3.68** | **429** | **20** | **2** | **18.78 ± 0.98** | **5.21** | **641** |  |
| VS | 71 | 6 | 22.3 | 74 | 26 | 3 | 4.45 ± 0.18 | 4 | 12 | 222 | 16 | 2 | 15.18 ± 0.64 | 4.21 | 440 | 20 | 2 | 19.48 ± 0.45 | 2.31 | 634 |  |
| VS | 175 | 6 | 25 | 186 | 21 | 2 | 4.50 ± 0.17 | 3.8 | 10 | 217 | 5 | 1 | 14.21 ± 0.27 | 1.9 | 440 | 21 | 1 | 18.37 ± 0.53 | 2.87 | 626 |  |
| VS | 176 | 7 | 25 | 188 | 16 | 1 | 4.93 ± 0.33 | 6.7 | 10 | 217 | 15 | 1 | 13.86 ± 0.50 | 3.64 | 383 | 22 | 1 | 18.73 ± 0.90 | 4.78 | 639 |  |
| VS | 180 | 6 | 25 | 185 | 63 | 3 | 4.44 ± 0.18 | 4 | 10 | 217 | 31 | 2 | 13.20 ± 0.50 | 3.79 | 391 | 20 | 1 | 18.55 ± 0.82 | 4.41 | 655 |  |
| VS | 182 | 5 | 23 | 187 | 57 | 2 | 4.24 ± 0.18 | 4.2 | 10 | 217 | 16 | 1 | 12.94 ± 0.71 | 5.47 | 391 | 26 | 1 | 17.33 ± 0.99 | 5.73 | 655 |  |
| VS | 184 | 6 | 24.2 | 187 | 16 | 1 | 4.61 ± 0.17 | 3.6 | 10 | 217 | 7 | 1 | 14.29 ± 0.38 | 2.63 | 403 | 22 | 1 | 19.07 ± 0.76 | 4 | 639 |  |
| VS | 190 | 6 | 27.5 | 195 | 62 | 2 | 4.63 ± 0.18 | 4 | 10 | 220 | 32 | 1 | 13.82 ± 0.92 | 6.68 | 398 | 20 | 1 | 18.46 ± 0.74 | 4.01 | 639 |  |
| VS | 194 | 5 | 24 | 195 | 16 | 1 | 4.81 ± 0.28 | 5.8 | 10 | 217 | 14 | 1 | 15.20 ± 0.66 | 4.35 | 416 | 25 | 1 | 19.57 ± 0.89 | 4.56 | 626 |  |
| **Data in bold was used for degree days (DD) as collected by the same person.** | | | | | | | | | | | | | | | | | | | | |  |

Egg size (diameter, mm; ± standard deviation) was taken from measurements at both post-fertilisation (PF) and eye stage (E) for each family (i.e. two measurements per family.

CV, coefficient of variation; N, sample size; #cages, number of cages sampled per developmental stage; DD, degree days. FL, fork length (cm); SL, standard length of embryos (mm).
